# Stress response to CO_2_ deprivation by *Arabidopsis thaliana* in plant cultures

**DOI:** 10.1371/journal.pone.0212462

**Published:** 2019-03-13

**Authors:** Souvik Banerjee, Oskar Siemianowski, Meiling Liu, Kara R. Lind, Xinchun Tian, Dan Nettleton, Ludovico Cademartiri

**Affiliations:** 1 Department of Materials Science & Engineering, Iowa State University of Science and Technology, Ames, IA, United States of America; 2 Department of Statistics, Iowa State University of Science and Technology, Ames, IA, United States of America; 3 Bioinformatics and Computational Biology Program, Iowa State University of Science and Technology, Ames IA, United States of America; 4 Department of Chemical & Biological Engineering, Iowa State University of Science and Technology, Ames, IA, United States of America; 5 Ames Laboratory, U.S. Department of Energy, Ames, IA, United States of America; National Research Council of Italy, ITALY

## Abstract

After being the standard plant propagation protocol for decades, cultures of *Arabidopsis thaliana* sealed with Parafilm remain common today out of practicality, habit, or necessity (as in co-cultures with microorganisms). Regardless of concerns over the aeration of these cultures, no investigation has explored the CO_2_ transport inside these cultures and its effect on the plants. Thereby, it was impossible to assess whether Parafilm-seals used today or in thousands of older papers in the literature constitute a treatment, and whether this treatment could potentially affect the study of other treatments.For the first time we report the CO_2_ concentrations in Parafilm-sealed cultures of *A*. *thaliana* with a 1 minute temporal resolution, and the transcriptome comparison with aerated cultures. The data show significant CO_2_ deprivation to the plants, a drastic suppression of photosynthesis, respiration, starch accumulation, chlorophyll biosynthesis, and an increased accumulation of reactive oxygen species. Most importantly, CO_2_ deprivation occurs as soon as the cotyledons emerge. Gene expression analysis indicates a significant alteration of 35% of the pathways when compared to aerated cultures, especially in stress response and secondary metabolism processes. On the other hand, the observed increase in the production of glucosinolates and flavonoids suggests intriguing possibilities for CO_2_ deprivation as an organic biofortification treatment in high-value crops.

## Introduction

Thousands of papers each year (~14500 since 2014[1]) use plant cultures in Petri dishes–seeds (typically of *Arabidopsis thaliana*) germinated in sealed, square, vertically held, gel plates–as a model system. The simplicity, throughput, frugality, practicality, transparency, and sterility of this protocol has made it widely adopted to study the biology of plant development (e.g., root formation[2]), stress response (e.g., drought[3]), and interactions with other organisms (e.g., rhizosphere interactions[4, 5]). In order to avoid drying and contamination, these cultures are sealed usually by a film of paraffin (commercially known as Parafilm) or porous tape (commercially known as Micropore tape).

Parafilm was the recommended seal for *A*. *thaliana* cultures as recently as five years ago[6], and was nearly universally used in earlier studies that are referenced and studied today. A significant portion of the experimental plant biology community is not aware of the extent of stress that Parafilm seals can induce on plant cultures[7–9]. Parafilm-sealed cultures are still widely used in high profile publications and by prestigious laboratories, and are still the standard tool to study volatile-based plant-microbe interactions *in vitro*[4, 10, 11]. The “aeration issue” is often thought to be effectively mitigated by shorter term cultures or by focusing on pathways that are not directly associated with photosynthesis. Remarkably, the magnitude of the effects of Parafilm seals on transcription and phenotypes, and on the dynamics of CO_2_ uptake and release by *A*. *thaliana* cultures has not been reported until now.

This characterization of the phenotypic and transcriptomic response to CO_2_ deprivation in *A*. *thaliana* could be valuable for three reasons: (i) reevaluating old and new reports that use Parafilm-sealed *A*. *thaliana* cultures, where CO_2_ deprivation could modify the effects of the same treatments in less stressful environments; (ii) understanding the biological response of plants to CO_2_ deprivation[12, 13] that is observed, for example, in crop canopies[14, 15]; (iii) assessing the effects of Parafilm-seals on modern studies of volatile-based microbe-plant interactions[4, 5]; (iv) obtain a better understanding of plant response to CO_2_ scarcity in the past[16, 17]. In this work, we have engineered square Petri dishes (10 cm×10 cm) for active aeration (described in the Materials and Methods section) of *A*. *thaliana* plant cultures and compared them with Parafilm and Micropore tape wrapped plant cultures (plant cultures in Petri dishes were kept in an upright position) for characterization of stress response to CO_2_ deprivation.

## Results and discussion

**Fig 1:**
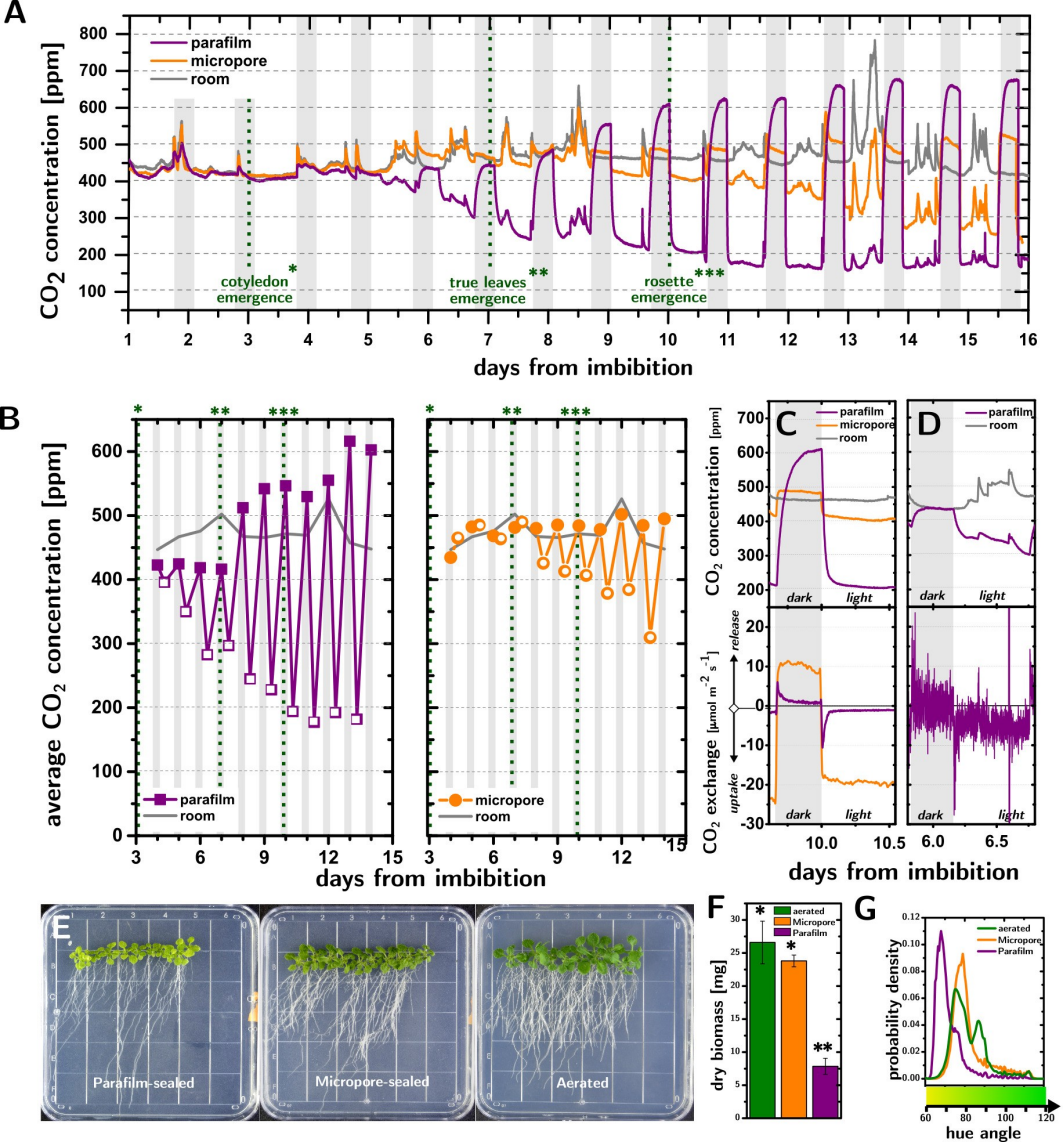
CO_2_ deprivation in Petri dish cultures of *A*. *thaliana*. **A.** Time evolution of the CO_2_ concentrations in Parafilm-sealed (purple) and Micropore-sealed (orange) Petri dishes containing 15 *A*. *thaliana* (Col-0) plants, compared to room concentration (grey). **B.** CO_2_ concentrations averaged over the light periods (empty points) and dark periods (filled points). **C.** CO_2_ concentration (top plot) and CO_2_ exchange rates (bottom plot) at day 10 in Parafilm-sealed and Micropore sealed dishes. The Parafilm-sealed dishes experience severe suppression of both photosynthetic and respiratory rates, as compared to Micropore-sealed dishes. White and grey background shadings indicate light and dark periods, respectively. Different number of asterisks indicate three stages of development namely cotyledon emergence, true leaves emergence, and rosette emergence. **D.** CO_2_ concentration (top plot) and CO_2_ exchange rates (bottom plot) at day 6 in Parafilm-sealed dishes. The mild depletion of CO_2_ seen in the top plot, is still accompanied by severe suppression of photosynthetic CO_2_ uptake and respiration (bottom plot). **E.** Photographs of Parafilm-sealed, Micropore-sealed, and aerated plates. **F.** Dry biomass. Different numbers of asterisks above the histograms indicate a significant difference (P<0.05, Students t-test) between the treatments. **G.** Hue distribution for the leaves of *A*. *thaliana* cultures in Parafilm-sealed, Micropore-sealed, and aerated plates.Probability density indicate the probability density function of the hue angle variable (obtained using the “ksdensity” function in Matlab with a bandwidth of 0.8).

Photosynthesis and respiration strongly modify the concentration of CO_2_ ([CO_2_]) inside Petri dish cultures of *A*. *thaliana* (15 plants per dish, 0.5 Murashige-Skoog nutrient medium with 1% by weight of sucrose[18]). Fig 1A shows that the [CO_2_] inside the dishes undergoes very significant changes at the onsets of light and dark periods, which increase in magnitude with the development of the plants.

When using Parafilm seals, the [CO_2_], averaged over the light period (Fig 1B, open purple squares), decreases from 396 ppm to 177 ppm between the emergence of the cotyledons (day 4) and the emergence of the rosettes (day 11), after which it stops decreasing (RuBisCO binds preferentially to O_2_ when [CO_2_]<200ppm, preventing carbon fixation[19]). With Micropore seals instead, the depletion of the [CO_2_] averaged over the light period (Fig 1B, open orange circles) becomes only evident when the first true leaves emerge (day 7), after which it decreases by approximately 26 ppm per day to reach 305 ppm at day 14. Respiration increases significantly the [CO_2_] in the cultures during the dark periods (Fig 1B, filled points), especially with Parafilm seals, where [CO_2_] can reach values higher than 600 ppm. While these changes in [CO_2_] are a function of the number of plants and their age, we observed that even 5 plants cause [CO_2_] to decrease below 200 ppm at day 11 (S5A Fig) in Parafilm-sealed dishes.

Importantly, the respiration of the experimenters (cf. day 13 in Fig 1A) caused significant fluctuations of the [CO_2_] in the cultures, especially in those sealed with Micropore tape.

Measurements of [CO_2_] with 60 s time resolution provide insight into the kinetics of CO_2_ uptake and release by the cultures and how they are affected by the seals. The top panel of Fig 1C compares the [CO_2_] changes in Micropore- and Parafilm-sealed cultures in response to the transition between light and dark periods at day 10. [CO_2_] decreases and increases rapidly as the light is switched on and off (as fast as -6.0 ppm/min and +3.7 ppm/min), but these changes slow down less than 2 hours after each transition.

The different shape of the [CO_2_] traces obtained for the different seals suggest that CO_2_ exchange by the plant could be affected by CO_2_ deprivation. By independently measuring the effective diffusivity of the seals to CO_2_ and the area of the leaves vs time, we were able to estimate the total exchange of CO_2_ by the plants (i.e., the μmoles exchanged by 1 m^2^ of leaf area per unit second, J_plant_, here defined as the sum of the photosynthetic and respiration rates, J_photos._<0 and J_resp._>0, respectively) as a function of time (Fig 1C, bottom panel).

The results from the two seals are strikingly different. In Micropore-sealed cultures, J_plant_ reaches a steady state (10±1 μmol·m^-2^·s^-1^ in dark and -19.3±0.9 μmol·m^-2^·s^-1^ in light) within 20 minutes of the light turning off and on. With Parafilm seals, the exchange of CO_2_ is instead strongly dependent on time: J_plant_ increases to a maximum value (8.19 μmol·m^-2^·s^-1^) within 3 minutes of the lights turning off, only to then decrease exponentially (R^2^ = 0.96, time constant = 44±1 min) by an order of magnitude (0.86±0.01 μmol·m^-2^·s^-1^). A strikingly similar behavior is observed during the light period, where J_plant_ reaches a maximum uptake value (-11.33 μmol·m^-2^·s^-1^) within 9 minutes of the lights turning on, only to reduce exponentially (R^2^ = 0.996, time constant = 28±1 min) by an order of magnitude (-1.080±0.003 μmol·m^-2^·s^-1^). The steady state value of J_plant_ during the light period and dark period for Parafilm-sealed dishes (-1.080±0.003 μmol·m^-2^·s^-1^ and 0.86±0.01 μmol·m^-2^·s^-1^) are 20 and 12 times lower than in Micropore-sealed dishes (-19.3±0.9 μmol·m^-2^·s^-1^ and 10±1 μmol·m^-2^·s^-1^), respectively, demonstrating a significant reduction of the photosynthetic rate and metabolism in Parafilm-sealed dishes.

Remarkably, the low CO_2_ exchange observed in Parafilm-sealed dishes occurs even when the [CO_2_] measured by the sensor is in a physiological, RuBP-regeneration-limited, range [19–21]. Fig 1D shows the [CO_2_] and J_plant_ for a Parafilm-sealed dish at day 6. While the [CO_2_] decreases from 433 ppm to 351 ppm as the light is turned on, uptake increases to a maximum (-27.7 μmol·m^-2^·s^-1^) only to decrease exponentially over time to a much lower value (-4.40±0.04 μmol·m^-2^·s^-1^). This result points to the formation of a CO_2_-depleted boundary layer[22] at the surface of the leaf in Parafilm-sealed dishes, which causes CO_2_ deprivation even when the bulk atmosphere is not depleted of CO_2_. This finding shows that early stages of Parafilm-sealed cultures are *not* protected from alterations to their CO_2_ metabolism.

Suboptimal levels of CO_2_ lead to leaf discoloration and stunted growth of *A*. *thaliana* in Parafilm-sealed dishes. Fig 1E shows *A*. *thaliana* (Col-0) cultures grown in Parafilm-sealed and Micropore-sealed dishes, compared to an aerated culture. Beside the difference in biomass (after three weeks, aerated cultures were 3.38 times larger than Parafilm-sealed cultures, p = 5·10^−4^, and not significantly larger than Micropore-sealed cultures, cf. Fig 1F), the hue distributions (Fig 1G) show that the aerated cultures are greener than the Micropore-sealed ones, which are much greener than the Parafilm-sealed cultures.

Because O_2_ levels in the Petri dishes are essentially constant in all the conditions we tested (S10 Fig), our first hypothesis was that the primary cause of these phenotypic changes is CO_2_ deprivation, which is expected to affect photosynthesis[23, 24], carbohydrate metabolism[25], and lead to photooxidative stress[26]. Further analysis of gene expression and metabolite production reveal a broad and systemic stress response in Parafilm-sealed dishes.

**Fig 2:**
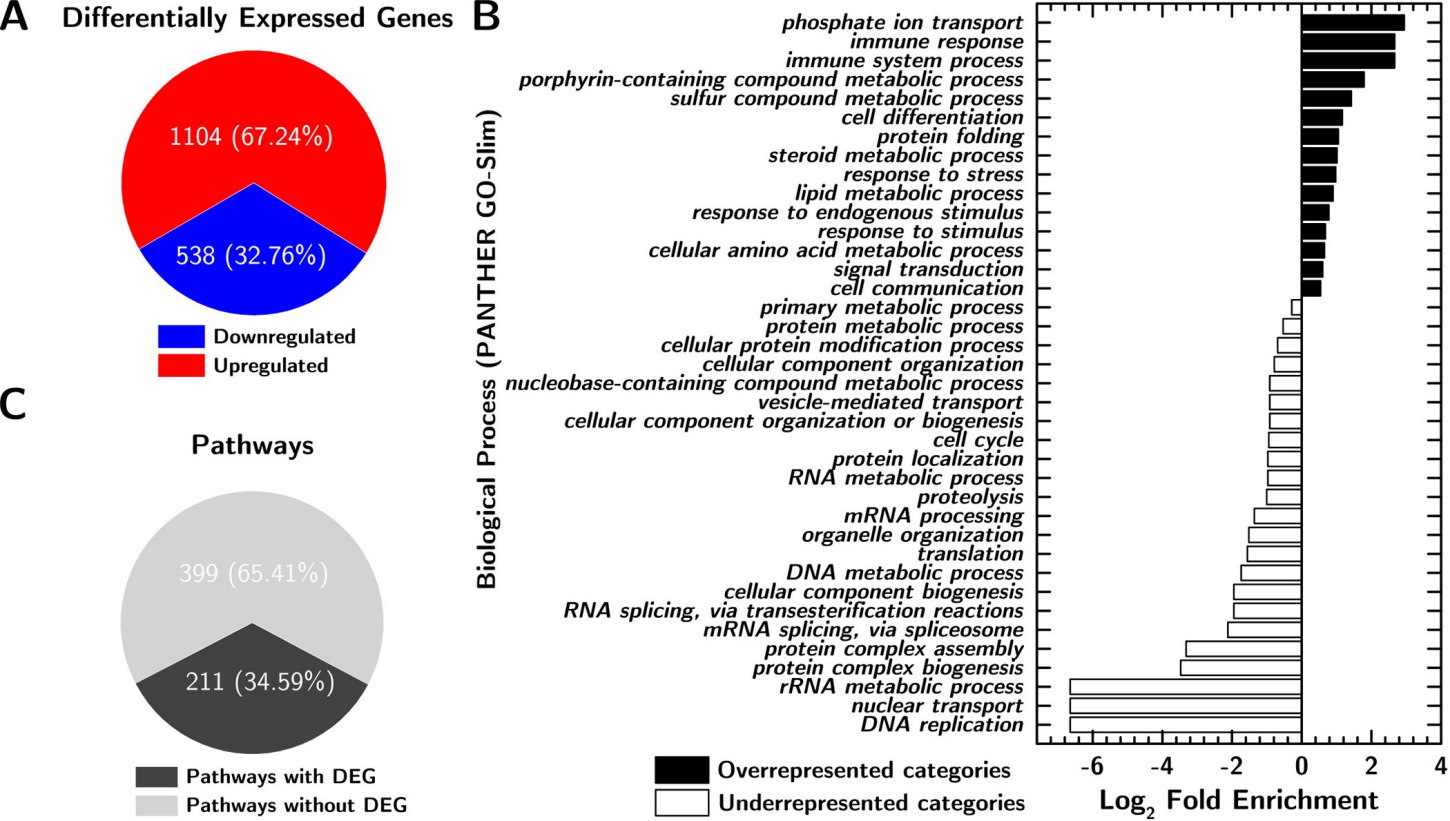
Gene expression analysis. **A.** Count and regulation of Differentially Expressed Genes (DEGs) in Parafilm-sealed plants compared to aerated plants. **B.** Gene Ontology enrichment analysis results for biological process. Panther analysis was made using Panther term enrichment tool version 11. **C.** Number of pathways significantly altered by culture in Parafilm-sealed dishes, compared to aerated conditions. Nonparametric multivariate analysis was performed to identify the Differentially Expressed (DE) gene categories for different pathways.

mRNA-Seq analysis conducted on both non-aerated and actively aerated Parafilm-sealed dishes (shoot samples collected at day 11, 8 hours after the beginning of the light period) showed a significant (Benjamini-Hochberg adjusted p value < 0.05[27] and fold change ≥ 2) alteration of gene expression (Fig 2A) caused by Parafilm seals (1642 differentially expressed genes (DEGs), 67% of which were upregulated) when compared to aerated dishes. Only three genes, At4g06746 (AtDEAR5, DREB subfamily A-5 of ERF/AP2 transcription factor family), At3g29035 (AtORS1, protein with transcription factor activity, the mRNA is cell-to-cell mobile), and At3g02550 (lateral organ boundaries (LOB) domain protein 41 (LBD41)) were upregulated by growing plants in Micropore sealed dishes when compared to actively aerated dishes, indicating relatively little impact of the [CO_2_] oscillations caused by Micropore seals (at least at day 11). Neither of the upregulated genes can be directly connected to plant response to potentially limiting CO_2_ concentration but all three DEGs have been shown to change upon abiotic (drought, salt, heat, hypoxia) and biotic stresses[28–30]. This might indicate that the plants we grew in Micropore sealed dishes were under mild stress (an increase in the biomass, either due to more or larger plants would then increase this stress level).

A Gene Ontology enrichment analysis (Fig 2B) showed significant overrepresentation of stress response and secondary metabolism processes and underrepresentation of primary metabolism and molecular mechanisms such as DNA or RNA processing among DEGs. Nonparametric metabolic pathway analysis (Fig 2C) revealed that 211 out of 610 metabolic pathways were significantly altered (false discovery rate, FDR, < 0.05). Among these altered pathways were those for carbohydrate metabolism, chlorophyll biosynthesis, secondary metabolites biosynthesis, and stress response.

**Fig 3:**
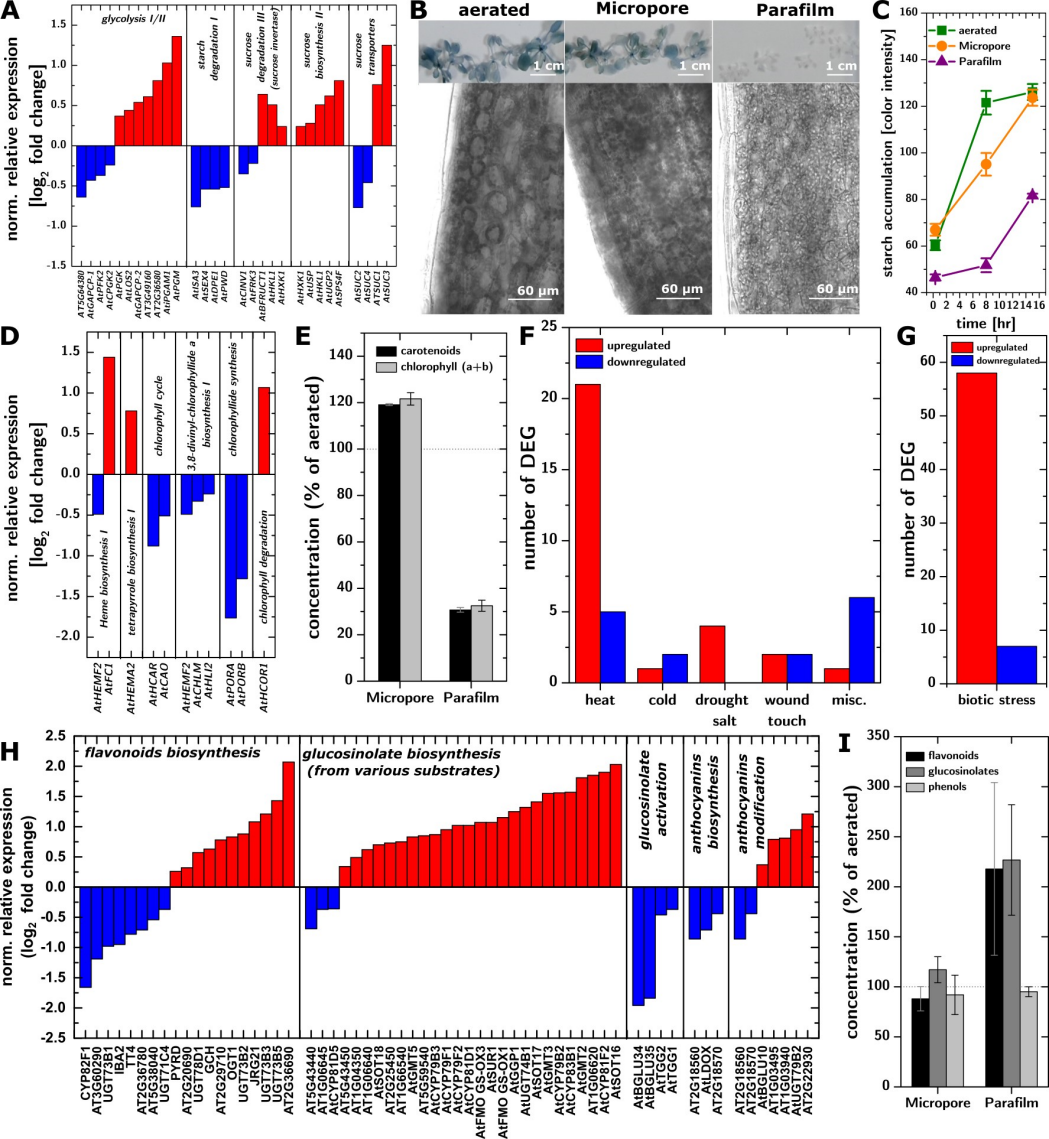
Plant molecular and metabolic phenotypes. **A.** DEGs in pathways related to sugar metabolism, indicating upregulation of glycolysis and sucrose metabolism and downregulation of starch degradation. **B.** Accumulation of starch in leaves (whole plant colorimetric assay and 10x micrographs) in aerated (left), Micropore-sealed (middle), and Parafilm-sealed (right) plants. C. Time-resolved starch accumulation quantification during the light period in aerated (green squares), Micropore-sealed (orange circles), and Parafilm-sealed (purple triangles) plants. Lines are guides to the eye. **D.** DEGs on pathways related to chlorophylls metabolism, showing downregulation of the chlorophyll biosynthesis pathways. **E.** Chlorophyll concentration in Micropore-sealed and Parafilm-sealed dishes (% of the aerated plants). Error bars represent standard error (95% CI, n = 3 replicates /10 plants each). **F-G.** Number and regulation of DEGs associated with abiotic or biotic stresses. **H.** DEGs in pathways associated with the synthesis of glucosinolates and flavonoids. **I.** Concentration (% of aerated plants) of flavonoids, glucosinolates and phenolic compounds. Error bars represent standard error (95% CI, n = 3 replicates /10 plants each).

The simultaneous upregulation of glycolysis and sucrose metabolism and downregulation of starch degradation suggested that the carbohydrate metabolism was compromised (Fig 3A) and that sucrose had replaced CO_2_ as a carbon source. Parafilm-sealed cultures showed minimal starch accumulation throughout the entire light period (Fig 3B and 3C), which demonstrates that the plants struggled to produce excess photosynthate, consistently with the lack of CO_2_ substrate. The lack of accumulated starch is also consistent with the low rates of respiration observed during the dark period in Parafilm-sealed cultures (Fig 1C). Micropore-sealed cultures accumulate starch faster than Parafilm-sealed cultures, but slower than aerated cultures. Parafilm-sealed dishes also led to downregulation of the chlorophyll biosynthesis pathways (Fig 3D) and lower concentrations of chlorophyll (Fig 3E), while Micropore-sealed dishes did not affect chlorophyll production significantly. Plants grown in Parafilm-sealed dishes showed a decreased concentration of carotenoids (proportionally to chrolophylls). This might be due to inhibited metabolisms caused by lack of carbohydrates in non-aerated plants. However, decreased carotenoids concentration could be the result of a low concentration of chlorophylls. Light harvesting by less chlorophylls could result in slower generation of chlorophyll triplet states or/and singlet oxygen that carotenoids protect from[31].

Upregulation of genes associated with biotic and abiotic stress (Fig 3F–3G) is consistent with the hypothesis that CO_2_ deprivation resulted in a systemic stress response, potentially due to the accumulation of reactive oxygen species (ROS)[32]. We observed a upregulation of SuperOxide Dismutase 1 and 2 (SOD1 and SOD2) encoding enzymes that catalyze the dismutation of superoxide radicals into H_2_O_2_ [33, 34].

A potential silver lining of these findings lies in the secondary metabolite biosynthesis, which is mostly upregulated in Parafilm-sealed cultures. In the case of flavonoids and glucosinolates, this upregulation (Fig 3H) is matched by an increased concentration of these species in the plants (Fig 3I). Glucosinolates are under intense investigation for potential medicinal uses[35, 36], and flavonoids are powerful antioxidants[37] that are important in the interaction of plants with their environment[38] and are partly responsible for flavor and aroma of fruits and vegetables[39]. In light of these results, controlled CO_2_ deprivation–a relatively easily scalable and organic treatment–should be considered a candidate treatment for increasing the production of high value medicinal compounds and boosting or tuning the flavor of high value crops (e.g., strawberries, grapes).

In conclusion it might be helpful to use these data to provide some recommendations on experimental procedures that would avoid CO_2_ deprivation in *A*. *thaliana* cultures in Petri dishes. 1. Do not use the data in this paper to estimate how many plants you could grow in Parafilm-sealed dishes without incurring into CO_2_ deficiency. The data in Fig 1D clearly show that CO_2_ deficiency occurs as soon as the leaves emerge. As those data show, the [CO_2_] concentration measured by sensors in the dish do not represent the state of CO_2_ deficiency at the leaf. In the case of Micropore seals, our data do not allow to estimate accurately at what rate of collective CO_2_ uptake the Micropore-sealed cultures will also incur in significant CO_2_ depletion. Therefore, until more complete measurements are conducted on the limits of Micropore-sealed cultures, we would recommend to use them within the parameters (i.e., number of plants and duration of culture) explored here.

2. Whether you decide to use Parafilm or Micropore seals, always explicitly report it in your papers so that your data could be better understood in light of our growing understanding of CO_2_ deprivation. In the absence of that information it might be difficult in the future to fully understand your conclusions. Also, importantly, report how many wrappings of seal do you use.

3. If you introduce a new seal, characterize in detail the [CO_2_] inside the cultures with a high time resolution (1 min or less) to allow the visualization of the difference in [CO_2_] between light and dark periods, and to allow the characterization of the rates of uptake and release. Quantification of the rates of uptake and release is essential to assess the effect on the plant. If possible, also conduct a transcriptome analysis.

4. As new technologies emerge that will allow the facile aeration of cultures, use them in case you need to grow plants in vitro for extended periods.

## Materials and methods

### Plant material

*Arabidopsis thaliana (A*.*thaliana)* (ecotype Col-0) was chosen as a model organism for all studies in Petri dishes using the sterile approach described by Xu et. al. and Lindsey et. al.[40, 41] with modifications. Briefly, surface sterilized seeds (first, soaked in 70% ethanol for 1 minute; then immersed in 1:5 v/v sodium hypochlorite solution with 0.05% surfactant (Tween 20) for 8 mins; last, washed 6 times with sterile H_2_O) were planted on a half strength Murashige and Skoog with 0.8% agar and 1% w/v sucrose. The pH was adjusted to 5.7–5.8 in square Petri dishes (dimensions 10 cm×10 cm, FisherBrand, USA) under sterile conditions.[42, 43] The plant cultures were wrapped and sealed with either paraffin films (commercially known as Parafilm) or tapes (commercially known as Micropore tape). The plants were grown in a 16h day/8h dark cycle with ambient temperatures of 21±3°C with light intensity [130–160 μmol m^-2^ s^-1^] under LED light.

### Measurement of CO_2_ concentration

#### CO_2_ sensor setup

All CO_2_ concentration was measured with NDIR CO_2_ sensors (K30 module from Senseair purchased from CO_2_ meter.com) with a sensitivity range of 0–5000 ppm within specifications and an accuracy of ±30 ppm (±3% of measured value within specifications) powered at 5V with an Arduino Uno microcontroller (S1 Fig). Data were acquired with Arduino assembled data logging shield (Adafruit item #1141) using a SD card. The output of the sensor is the CO_2_ concentration (ppm by volume) and time in seconds. Data were collected every second from the sensor using an arduino code.

#### Calibration of CO_2_ sensors

The CO_2_ sensors were calibrated with standard gases of known CO_2_ concentrations (0, 100, 500, 1000 ppm standards) as input and the output reading of the sensors were recorded. The fitted equations were then used to calibrate all the raw data from the sensors. All the sensors behaved linearly within this range. Calibration curves of CO_2_ sensors used to measure CO_2_ concentrations in the room and in Parafilm and Micropore wrapped plant cultures in Fig 1A are shown in S2 Fig with the fitting parameters shown in S1 Table. Calibration data for all sensors used in CO_2_ characterization experiments are available in S4 Supplementary data.

#### Incorporation of CO_2_ sensor into Petri dish

The main limitation of measuring CO_2_ in real time (every second) is the small volume of Petri dish (~106 ml) and the requirement of a sterile, closed environment. The CO2 sensor from Senseair fits the size of Petri dish and could be embedded in the Petri dish without blocking light or touching the gel surface (S3 Fig). To fit the sensor on the top cover of the Petri dish, the outline of the sensor membrane surface was created by melting the top cover using a soldering iron in the biosafety cabinet. The sensor/Petri dish contact area was further sealed with autoclaved soft silicone putty (Ear Mack’s product, Walmart # 004055880) to avoid any leaks. The set up can be surface sterilized with ethanol inside the bio-safety cabinet without comprising gas measurement.

### Aeration for Petri dish

#### Aeration system setup

The aeration system was made with a screw cap Erlenmeyer flask (tightly screwed to avoid any loss of air flow) attached to a silicone tubing of (1/4ʺ ID, 3/8ʺ OD, McMaster-Carr #5236K87 and 1/8ʺ ID, 1/4ʺ OD McMaster-Carr #51135K15) connected to a double outlet aquarium pump (Walmart item #000816269). The set up was then attached to the inlet of the Petri dish. The flow was divided using a T-separator in such a way that four Petri dishes can be aerated with one flask. The minimum flow rate was found in the range of 0.15–0.25 lpm.

#### Incorporation of aeration system into Petri dish

The Petri dishes were engineered for active aeration (S4 Fig). The tips of sterile syringe filters (mixed cellulose esters, 0.22 μm pore size, 33 mm diameter, Fisher Scientific item # 09-720-004) were wrapped with stretched sterile Parafilm strips (sterilized in a 70% ethanol bath for 1–2 hours). A soldering iron was used in the biosafety cabinet to burn through the top of Petri dish to create holes for inlet and outlet and the Parafilm wrapped filters were mounted. The Petri dish with filters was placed in a sterile box and kept for 1–2 hours at 70–80°C in the oven. The Petri dishes were moved from oven to biosafety cabinet, and the filters were twisted and pressed into the holes to seal gaps at the connections.

### Characterization of CO_2_ trace

CO_2_ concentrations were measured simultaneously inside and outside of Petri dishes sealed with Parafilm or Micropore tape and containing 5 or 15 *A*. *thaliana* plants (S5 and S6 Figs). The plates were kept for vernalization at 4°C for 3 days after sowing the seeds. The gas measurement was started one day after vernalization in real time every 1 sec throughout the light and dark period. The sensor was switched on/off every ~24 hrs throughout the experiment to avoid any data loss due to overheating of sensors. The CO_2_ traces were measured until 19 days from plating. The collected data were averaged over 60 seconds to reduce noise. Time evolution of CO_2_ concentration was determined for all replicate experiments as shown in Table 1. CO_2_ trace of single replicates of Parafilm and Micropore-sealed plant cultures (each with 15 plants) is shown in Fig 1A. CO_2_ trace experiments were conducted, for all replicates, simultaneously under same conditions.

**Table 1:** Number of replicates per treatment for CO_2_ characterization within plant cultures.

Treatment (Type of membrane used to close the Petri dish w/ number of plants inside)	Number of Replicates
Micropore tape -15 plants	4
Parafilm—15 plants	3
Micropore tape—5 plants	5
Parafilm—5 plants	3

#### Measurement of dimensions of Petri dish

The dimensions of Petri dish used in calculations are listed in Table 2.

**Table 2:** Dimensions of Petri dish.

Parameters	Dimensions
Volume	0.00011 m^3^
Perimeter of the Petri dish	0.4 m
Width of the opening	0.001 m
Area of the width(gap between top and bottom plate)	0.0004 m^2^

#### Thickness determination of Parafilm and Micropore tape

Stretched Parafilm samples were frozen in liquid nitrogen. The imaging was done at a magnification of 20X using Nikon Eclipse upright microscope. The thickness was measured using ImageJ at multiple positions. SEM images of cross section of Micropore tape samples were taken at 150x magnification. The length of pores which span from top of the fiber to the adhesive side were considered for calculation. The average values and standard errors of thickness (95% CI) for Parafilm and Micropore tape are 46.9 μm±1.83μ*m*, 67.9μm±8.569μ*m* respectively.

#### Calculation of effective diffusivity of CO_2_ inside Petri dish sealed with Parafilm or Micropore tape

A tightly sealed box was used for this experiment. There is a sensor within the Petri dish and one outside. The source of CO_2_ (dry ice) was kept inside the box and the concentration vs. time profile was measured for Petri dishes sealed with Parafilm and Micropore tape respectively(S7 Fig). A fan was used for mixing the air inside the box to avoid inhomogeneities in the concentration of CO_2_. Both the sensors were placed next to the fan on the base. We started the measurement after evaporation of dry ice as the CO_2_ concentration inside the box has to be within the detection limit of the sensor. The Petri dish hosting the sensor was quickly placed inside the box after the dry ice evaporated.

The concentration vs. time plot can be made from the data collected from the experiment with a y-axis corresponding to CO_2_ concentration after de-dimensionalization and time as the x-axis. The concentration values in petri dish for multiple experiments was converted to dimensionless quantity using scaling method with the equation

$$\frac{C(t)-Cavg}{Cmax-Cavg}$$ (1)

Where C (t) is the concentration at each time point t, and C_avg_ is the overall average concentration, and C_max_ is the maximum concentration. The assumptions taken are as follows: 1. A constant source and finite sink for all the experiments. 2. The source is adequately mixed. 3. It is a one-dimensional diffusion problem. Petri dish is squared and symmetrical. So we can take four faces of Parafilm summed up as one membrane of defined thickness.

Fick’s first law of diffusion can be used to calculate the one dimensional diffusion problem (assuming mass flow remains constant) where J is flux in the unit of moles·m^-2^ ·s^-1^, D is the diffusion coefficient of the membrane for CO_2_ (m^2^·s^-1^), ΔC is the concentration difference between outside and inside (moles·m^-3^), Δx is the thickness of membrane (m). Now we know,

$$J=-D\frac{dc}{dx}$$ (2)

D/Δx is known as the permeability value of the membrane for CO_2_ and can be calculated from this experiment. Using the thickness (Δx) measured before we can measure the average D for all points along the curve and report the error values. The corresponding D values can be used to report the useful permeability values for both the membranes which can be used later for further calculations. We measured independently the diffusivity of CO_2_ through the seals to be D_eff_ = 1.0×10^−9^±6×10^−10^ m^2^·s^-1^ and 8×10^−08^±4×10^−08^ m^2^·s^-1^ for Parafilm and Micropore tape, respectively.

#### Measurement of leaf area

Images of the Petri dish were taken from the back side every day until the end of the experiment. They were then processed in ImageJ by color thresholding to convert the images to binary form. Then the total area of the leaves was plotted as a function of time. The data were then fitted with a power law and interpolated to obtain the leaf area as a function of time every 60 s. The interpolated data were then used to calculate the CO_2_ exchange rate discussed below.

#### Calculation of plant CO_2_ exchange rates

The rate of uptake and release were calculated using the following steps: 1) the concentration (ppm) data for the sensor within the Petri dish and the room sensor was converted to moles/m^3^ for inside and outside of petri dish. 2) The time was synced for each sensor in the Petri dish with the CO_2_ profile from room sensor. 3) The data were averaged every 60 sec. 4) In the first approximation the change in [CO_2_] with time is given by the sum of J_plant_·A_plant_ (where A_plant_ is the leaf area of the plants) and (D_eff_/Δx) ×Δ[CO_2_] where D_eff_ is the effective diffusivity of CO_2_, Δx is the thickness, and A_seal_ is the area of the seals, respectively, while Δ[CO_2_] is the difference in the [CO_2_] inside and outside the Petri dish. The uptake/release rates are calculated from the rate of concentration change within the Petri dish per unit time adjusted with the contribution of the membrane at each time point using the permeability values from the effective diffusivity experiment multiplied by Petri dish volume and normalized by measured leaf area for each day of measurement (S8 Fig).

### Measurement of O_2_ concentration

#### O_2_ sensor setup

The oxygen (O_2_) concentration inside the Petri dish was measured with LuminOx (LOX-02-S) optical oxygen Sensors (from sstsensing.com) with a sensitivity of 0–25% O_2_ and error of <2% powered at 5V with an Arduino Uno microcontroller (S9 Fig). Data were measured every second with an Arduino. Data were acquired in the same way as described above for the CO_2_ sensor.

#### Incorporation of O_2_ sensor into Petri dish

Each Petri dish was engineered for an O_2_ sensor in the same way as for a CO_2_ sensor. The outline of the sensor (sterilized with ethanol) was cut out of the Petri dish lid with a soldering iron inside the biosafety cabinet. The face of the sensor with the membrane was placed inside the outline in the Petri dish lid. The gaps between the sensor and the plastic of the lid were sealed with soft silicone putty as described before and left to dry inside the biosafety cabinet. The lid was then used as a regular Petri dish lid.

#### Characterization of O_2_ trace

Time evolution of O_2_ concentrations were measured inside and outside of Petri dish lined with Parafilm containing 10 *A*. *thaliana* plants (S10 Fig). The plates were kept for vernalization for 3 days at 4°C. The gas measurement was done following the same protocol described for the CO_2_ measurements.

### Phenotype analysis

For both aerated and non-aerated Petri dishes, the phenotypes were observed at the end of 3 weeks from imbibition for hue analysis as shown in Fig 1D and after 2 weeks out of fridge for the gene expression.

#### Experimental protocol

All experiments (secondary metabolite extraction/starch quantification) had three treatments: Aerated plant cultures, micropore and parafilm wrapped plant cultures. There were three replicates per treatment each having 10 plants inside. We did all the replicate experiments simultaneously in same conditions. We aerated all the Petri dishes with same flow rate using the method discussed earlier.

#### Secondary metabolite extraction

After the treatment, plants were photographed on Petri dish, flash frozen using liquid nitrogen and powdered in mortar. Powdered samples were kept at -80°C until use.

**Total glucosinolate:** The total glucosinolate content was measured following the method described by Mawlong et al. and Kestwal et al. [44, 45] with modifications. The glucosinolates were extracted using 80% methanol (HPLC grade, Sigma) from weighted flash frozen powdered samples (shoot) and kept overnight at room temperature. Next, the homogenate was centrifuged at 3000 rpm for 4 min and the supernatant was collected and diluted to 1 ml with 80% methanol. The reaction was conducted using: (i) 100 μl of the supernatant sample, (ii) 300 μl dH_2_O (distilled water) and 3 ml of 2 mM sodium tetrachloropalladate. The reaction mixture was incubated at room temperature for an hour and then its absorbance was measured at 425 nm. The absorbance of known concentrations of sinigrin was used as standard for quantification of total glucosinolates.

**Total flavonoids content:** The amount of flavonoids in each sample was determined by the aluminum chloride method described by Jia et. al.[46]. Samples were extracted following the method described by Sofo et al.[47] method using 100 μl of acidified methanol (1% HCl) for each 17 mg of frozen sample (powder). 500 μl of extract was then diluted with 500 μl of dH_2_O to which 150 μL of 5% sodium nitrite was added. Samples were vortexed and incubated at room temperature for 5 min. 150 μL of 10% aluminum chloride was added and samples were again vortexed and incubated at room temperature for 6 min. 2 mL of 4% sodium hydroxide was then added and samples were diluted to 5 ml with dH_2_O, vortexed and allowed to stand for 15 min at room temperature. Measurement of the absorbance was performed after the pink color developed due to the presence of flavonoids against the reagent blank at 510 nm. The amount of total flavonoids in the sample was expressed as mg rutin equivalents/g sample.

**Total Phenolic Content:** Total amount of phenolics in the samples was measured by using the Folin-Ciocalteu method described in Makkar’s manual [48]. Samples were extracted following the method described by Sofo et al. [47] using 100 μl of acidified methanol (1% HCl) for each 17 mg of frozen sample (powder). 50 μL of extract, 950 μl of dH_2_O and 500 μl of 0.5 mL of Folin-Ciocalteu reagent (1 N) were vortexed and allowed to stand for 5 min at room temperature. 2.5 mL of 5% sodium carbonate was added and the samples were vortexed again followed by incubation in the dark at room temperature for 40 min. Absorbance was measures after blue color developed against the reagent blank at 725 nm using spectrophotometer. The amount of total phenolics in the sample was expressed as mg gallic acid equivalents/g sample.

#### Detection of starch in plant material

The dynamics of starch accumulation was determined based on three-time points during the light period (two weeks after treatment) 20 minutes after the light is on, 8 hrs into the light period and 1 hour before the end of the light period for all the treatments. The rosettes were cut from each plant at the respective time points as mentioned before and placed in boiling water for 2 minutes to stop the plant metabolism. The rosettes were then placed in boiling absolute ethanol for 5 minutes. The boiling ethanol was changed with a fresh batch of ethanol and kept boiling for another 5 minutes. The ethanol was discarded with caution as the leaves are dehydrated and brittle and the vessel was filled with distilled water to allow the leaves to rehydrate. After 5 minutes images were taken of the bleached plants for each treatment. Bleached rosettes were then exposed to Lugol solution (1% KI/I) and left in the dark for 10 minutes. The samples were washed with distilled water to remove excess stain and placed on the Petri dish cover for final imaging. Calculation of starch accumulation was performed semiquantitatively by image analysis of pixel intensity. Photographs were made from the same perspective and under the same lighting (background intensity p-value = 0.58). Pictures were converted to 8 bit. Using ImageJ software, the average pixel intensity (i.e., the darker the pixel, the higher the concentration of dye and the higher is the pixel intensity) within each leaf of all plants in the experiment was measured (minimum of 10 plants per treatment with 3 replicates was used).

#### Detection of chlorophyll and carotenoids concentration

The chlorophyll and carotenoid content was measured using a previously described method.[49, 50] The samples were extracted using methanol and the concentration was assessed spectrophotometrically by analyzing absorption in a spectrum range from 449 nm to 700 nm.

#### Characterization of hue distribution across three treatments

The hue distributions of the leaves of Micropore-sealed, Parafilm-sealed and aerated samples was measured from photographs that were histogram matched to a reference image from the same experiment. The leaves were isolated by color thresholding in ImageJ. The RGB values for each pixel were extracted with Matlab and converted to HSV. The hue values, converted to hue angles, were then plotted as a distribution by using a kernel density plot.

### Genomic analysis

#### Treatments and experimental protocol

Two sets of mRNA sequencing was done. The need for two sets of mRNA sequencing data is attributed to the experimental design (to have a larger number of replicates to confirm our hypothesis).The first set was performed on two treatments: Parafilm-sealed plant cultures and aerated plant cultures. Both treatments had 4 replicates with 10 plants each. The second set of sequencing was performed for two treatments: Micropore-sealed plant cultures and aerated plant cultures. In this case both treatments had 4 replicates with 10 plants each.

#### RNA isolation

Two weeks after in vitro cultivation, with or without aeration, plants were photographed on Petri dish, flash frozen using liquid nitrogen and powdered in mortar. Total RNA was isolated from the samples for two treatments (Parafilm and aerated) using IBI Total RNA Mini Kit (Plant) according to the manufacturer’s instruction. RNA concentrations and integrity were determined using a RNA 6000 Nano Assay Kit and Bioanalyzer 2100(Agilent Technologies, Santa Clara, CA) with RNA 6000 ladder as the standards. Typical electropherograms and chromatograms of the total RNA suggested good quality of the RNA samples. Samples were stored at -80°C until use.

### Characterization of RNA-Seq data

The sequence archive from the ISU DNA facility website was extracted. FastQC v0.11.3 was used to determine the quality of the sequenced reads. All the sequence reads were determined to be of good quality, and no sequence trimming was necessary.

#### Genome and annotation

The reference genome (Arabidopsis_thaliana.TAIR10.dna.toplevel) was downloaded from http://plants.ensembl.org/info/website/ftp/index.html and the annotation/GFF file (Araport11_GFF3_genes_transposons.201606.gff.gz) from Araport11 website.

#### Mapping the reads to the reference genome

HISAT2 version 2.0.4, a splice-aware short read mapper, was used to map the reads to the reference genome using default settings. The aligned map files were then converted to sorted BAM files using samtools version 1.4.

#### Transcript abundance estimation

For quantifying gene transcript abundance from RNA-Seq data, we used featureCounts, version 1.5.2. The aligned reads were quantified against the gene features file (Araport11_GFF3_genes_transposons.201606.gff.gz), and the read counts for each gene transcript and each sample were written into a data matrix.

#### Differential expression analysis with DESeq2

DESeq2 normalizes the counts of genes by using median-of-ratios method[51], uses the counts of all genes to estimate dispersions and then employs a negative binomial model to calculate log fold change and p-values which are adjusted for multiple testing. The code is explained below.

#### Final curation

The results were filtered to exclude genes for which all samples showed zero counts. For gene descriptions of Arabidopsis, the biomart functionality of the plants ensembl website was used to get the gene descriptions/annotations which were appended to the working file.

#### Characterization of differently expressed pathways

Nonparametric multivariate analysis[52] was performed to identify the DE gene categories. First, the RNA-Seq counts were normalized by using upper-quartile normalization method of Bullard et. al.[53] and converted to log2-counts-per-million (logCPM). All genes were standardized to a common variance. Then, we computed the multiresponse permutation procedure test statistic (MRPP) and calculated the permutation p-value for each category. A significant threshold was chosen such that the false discovery rate (FDR) is maintained at a suitable level.

#### Characterization of Gene Ontology enrichment analysis for biological processes

Panther analysis was made using Panther term enrichment tool version 11.[54]

#### Characterization of differentially expressed genes associated with biotic and abiotic stresses

The differentially expressed genes associated with biotic and abiotic stresses was obtained using MapMan 3.6.0RC1 using TAIR 9 database.[55]

#### Gene expression data availability

The complete results of gene expression analysis (mRNA-Seq) and nonparametric pathway analysis are attached as tables in Excel file: S1 Supplementary Data (mRNA-Seq aerated to Parafilm) and S2 Supplementary Data (pathways analysis). The result of mRNA-Seq analysis of aerated and Micropore tape gene expression are shown in S3 Supplementary Data.

#### Accession numbers

The two sets of mRNA sequencing data are available through a linked repository (https://www.ncbi.nlm.nih.gov/geo/query/acc.cgi?acc=GSE120840) with the accession Number: GSE120840 and token no **ylcbmimmnhyxnqp** for the reviewers.

## Supporting information

S1 Fig Schematic of Arduino-sensor assembly. A. Connection schematic of CO_2_ sensor with Arduino. B. K-30 carbon-dioxide sensor module.
(TIF)

S2 Fig Calibration curves of CO_2_ sensors. Calibration curves of CO_2_ sensors used to measure CO_2_ concentrations in the room and in Parafilm and Micropore wrapped plant cultures (15 plants each). “Input” refers to the standard gases of different CO_2_ concentrations and “Output” refers to the sensor reading in response to individual input standard gases.
(TIF)

S3 Fig Schematic of CO_2_ sensor embedded inside the Petri dish. A. Pictogram of the engineered Petri dish with sensor inside B. Plants growing in engineered Petri dish with sensor after 2 weeks.
(TIF)

S4 Fig Petri dish modified for aeration with inlet and outlet ports as shown. (TIF)

S5 Fig Time evolution of CO_2_ concentrations in 5 plants. A. Micropore sealed Petri dishes with 5plants. B. Parafilm sealed Petri dishes with 5plants (dark period shown in gray) with multiple replicates (Petri dish cultures with 5 plants each).
(TIF)

S6 Fig Time evolution of CO_2_ concentrations in 15 plants. A. Micropore-sealed Petri dishes. B. Parafilm-sealed Petri dishes with 15 plants each (dark period shown in gray) with multiple replicates.
(TIF)

S7 Fig Characterization of the effective diffusivity of the membranes. A. Experimental set up. B. Concentration–time plot for two different membranes.
(TIF)

S8 Fig Dynamics of CO_2_ exchange. A Dynamics of CO_2_ exchange for plant cultures in Petri dishes. B Leaf growth dynamics over time for Parafilm and Micropore wrapped plant cultures with 15 plants.
(TIF)

S9 Fig Schematic of Arduino-sensor assembly of O_2_ sensor. (TIF)

S10 Fig Time evolution of O_2_ concentrations in Parafilm-sealed Petri dishes (red) containing 10 plants (Col-0) compared to room (black). (TIF)

S11 Fig Comparison of phenotypes for non-aerated and aerated plant cultures. **A** and aerated **B** plant cultures after 2 weeks before extraction of RNA samples for gene expression.
(TIF)

S1 Table Fitting parameters for the CO_2_ sensors used in the calibration curves in S2 Fig. (XLSX)

S1 Appendix Codes for Arduino sketches and DESeq analysis. (DOCX)

S1 Supplementary Data mRNA-sequencing data comparing aerated to parafilm. (XLSX)

S2 Supplementary Data Pathway analysis of aerated to parafilm. (XLSX)

S3 Supplementary Data mRNA-sequencing data comparing micropore to aerated. (XLSX)

S4 Supplementary Data Calibration data for CO_2_ sensors used in time evolution of CO_2_ concentration experiment. (XLSX)
