# Supplementary figures and images for: Stress response to CO2 deprivation by Arabidopsis thaliana in plant cultures
Source: PLoS One. 2019 Mar 13;14(3):e0212462. doi: 10.1371/journal.pone.0212462 (PMC6415875; doi:10.1371/journal.pone.0212462)

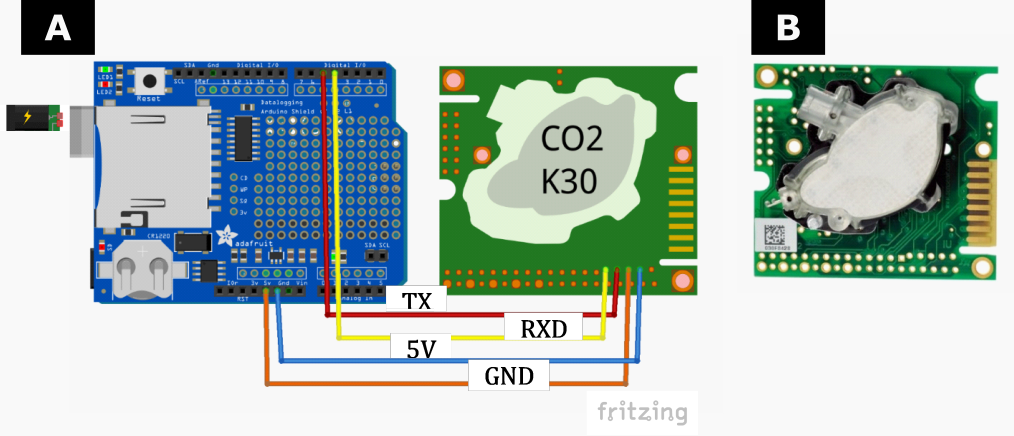

Supplement: S1 Fig — A. Connection schematic of CO2 sensor with Arduino. B. K-30 carbon-dioxide sensor module. (TIF) [file pone.0212462.s001.tif]

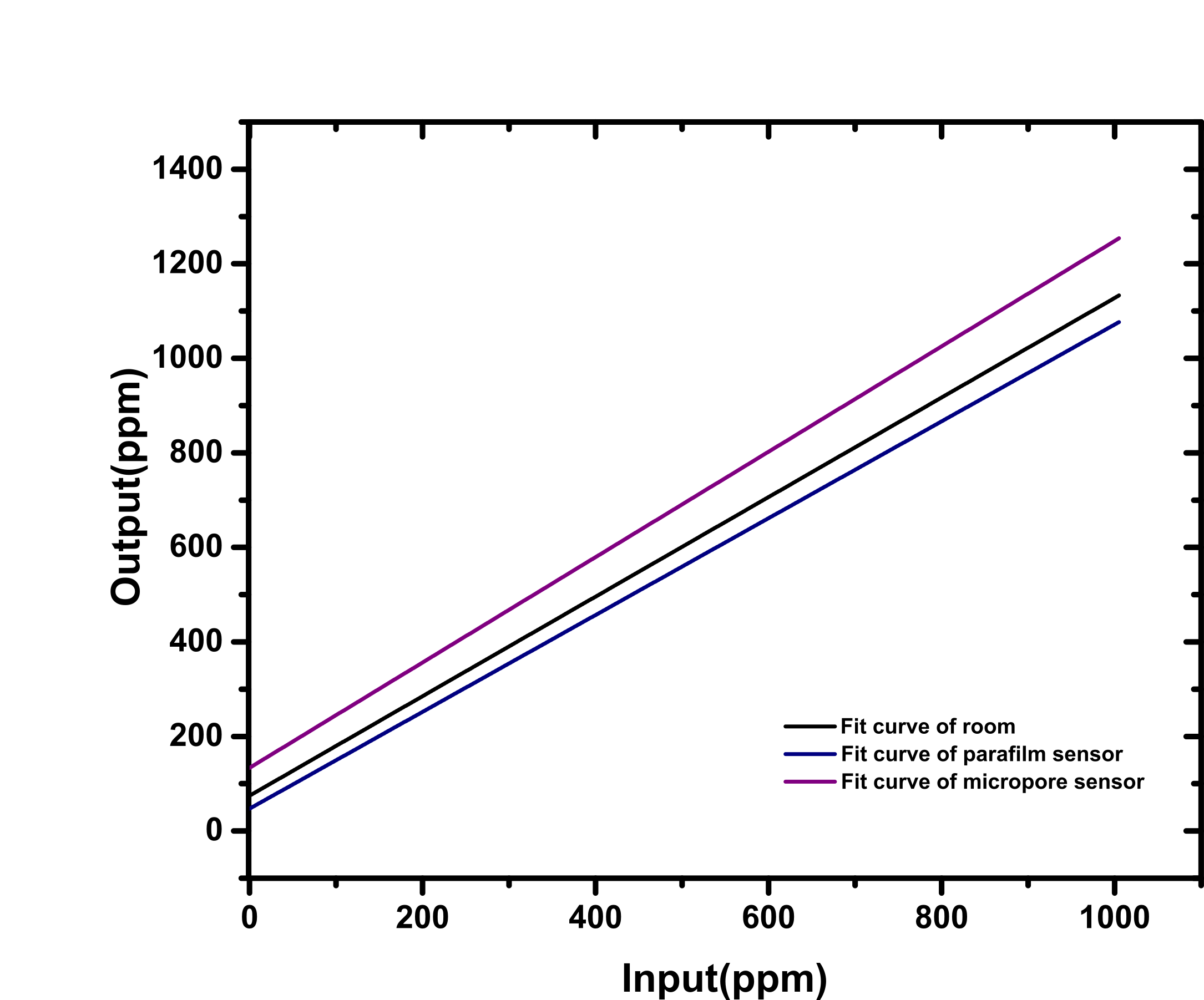

Supplement: S2 Fig — Calibration curves of CO2 sensors used to measure CO2 concentrations in the room and in Parafilm and Micropore wrapped plant cultures (15 plants each). “Input” refers to the standard gases of different CO2 concentrations and “Output” refers to the sensor reading in response to individual input standard gases. (TIF) [file pone.0212462.s002.tif]

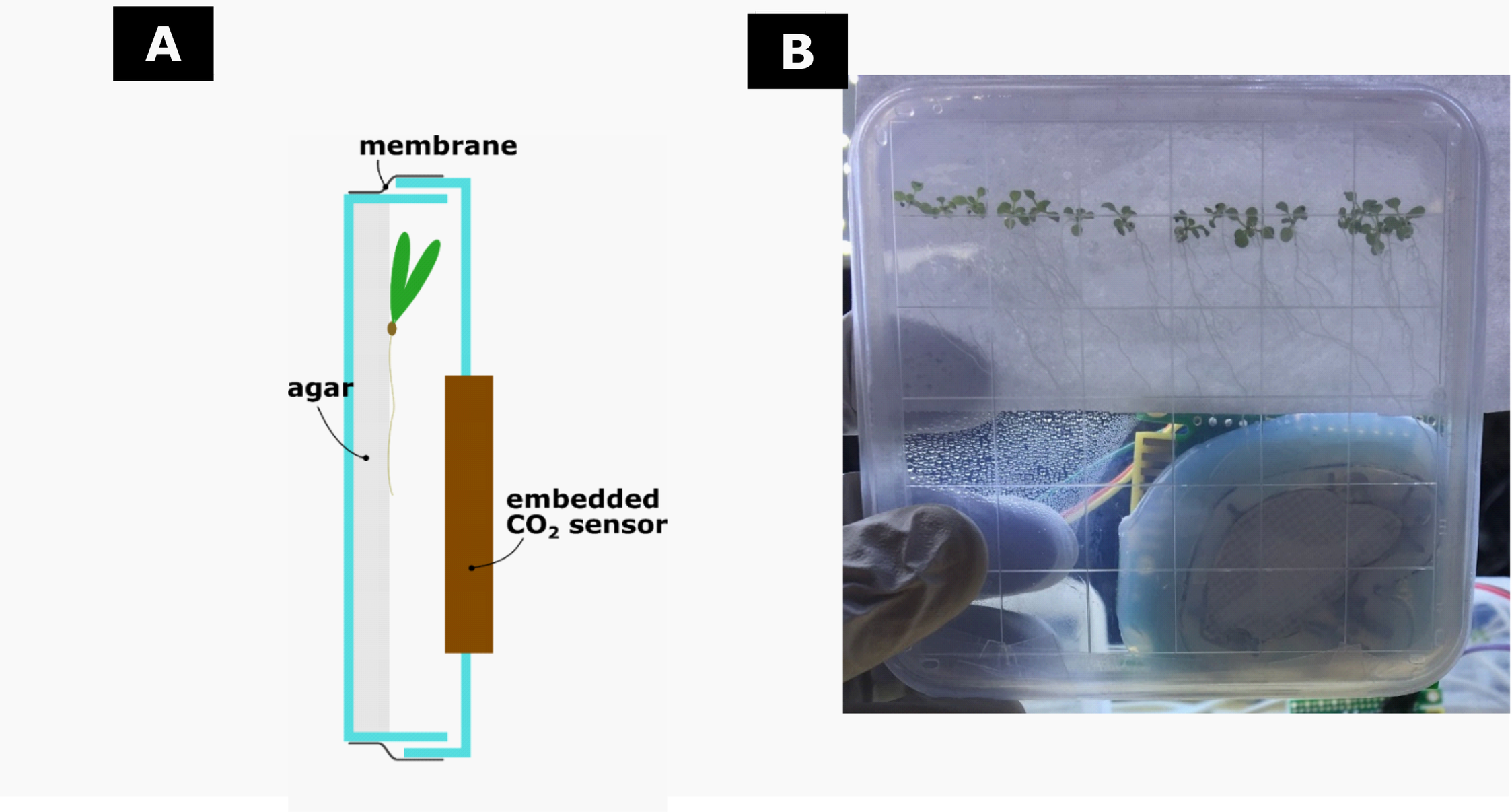

Supplement: S3 Fig — A. Pictogram of the engineered Petri dish with sensor inside B. Plants growing in engineered Petri dish with sensor after 2 weeks. (TIF) [file pone.0212462.s003.tif]

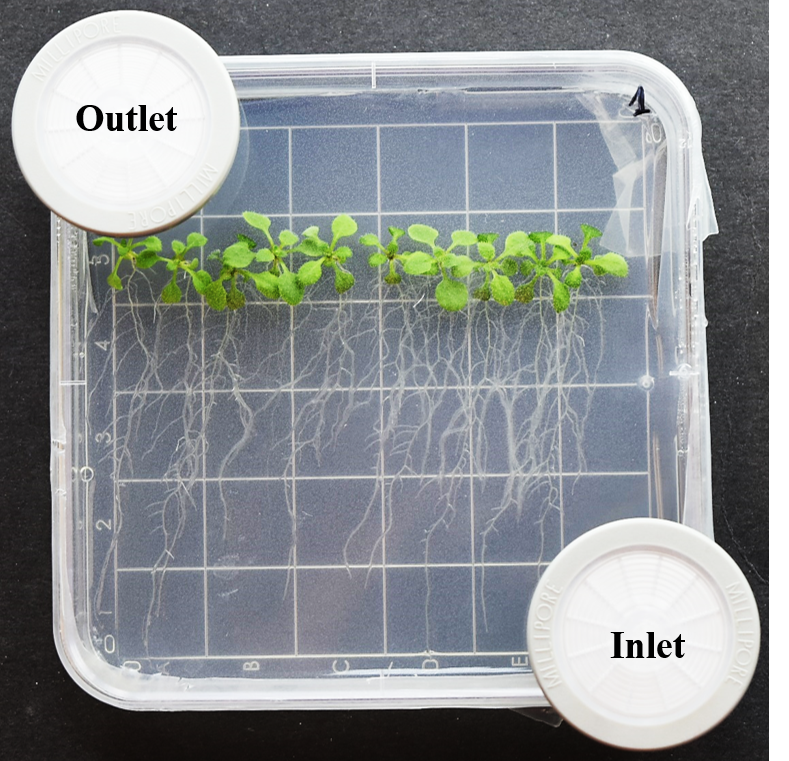

Supplement: S4 Fig — (TIF) [file pone.0212462.s004.tif]

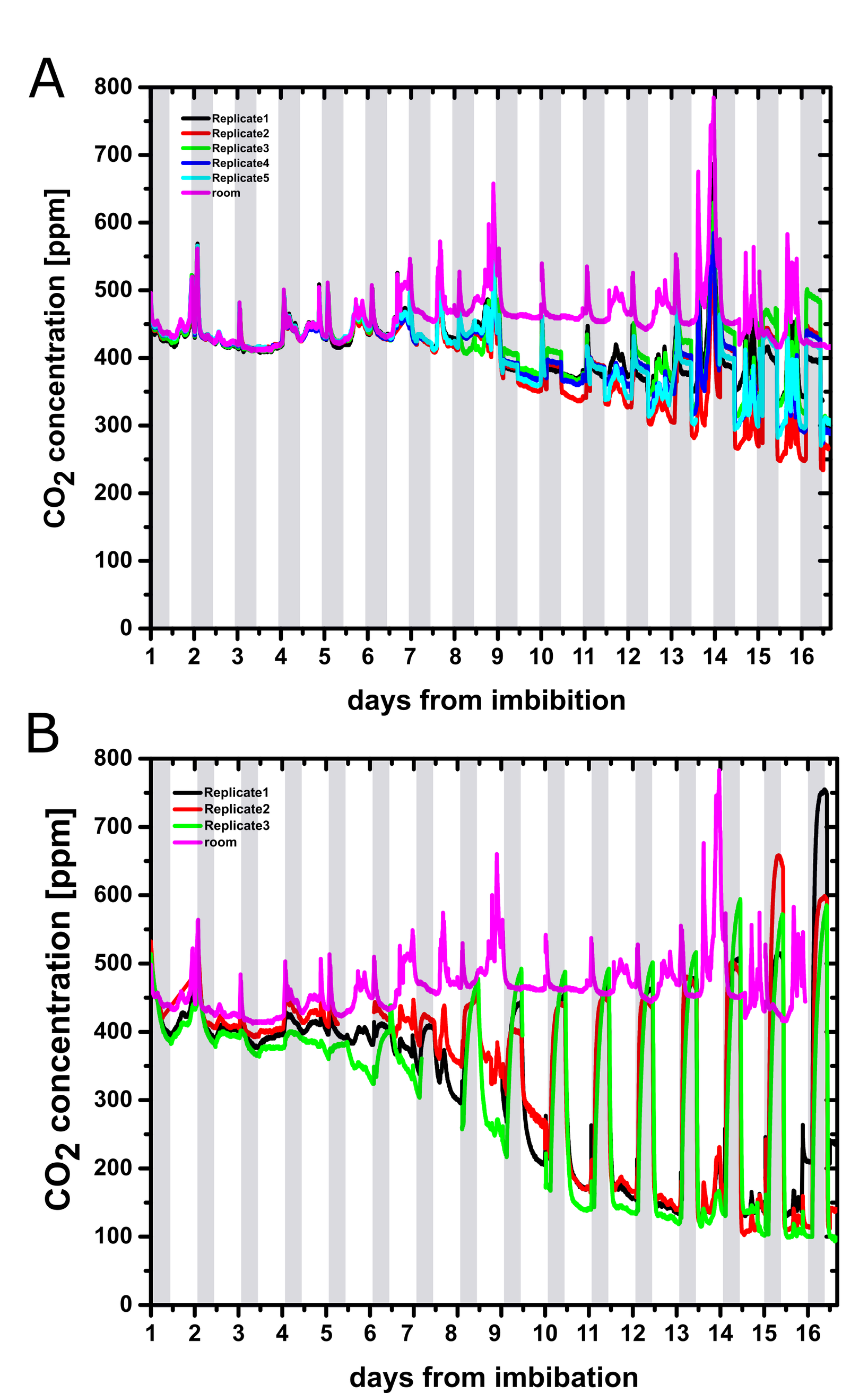

Supplement: S5 Fig — A. Micropore sealed Petri dishes with 5plants. B. Parafilm sealed Petri dishes with 5plants (dark period shown in gray) with multiple replicates (Petri dish cultures with 5 plants each). (TIF) [file pone.0212462.s005.tif]

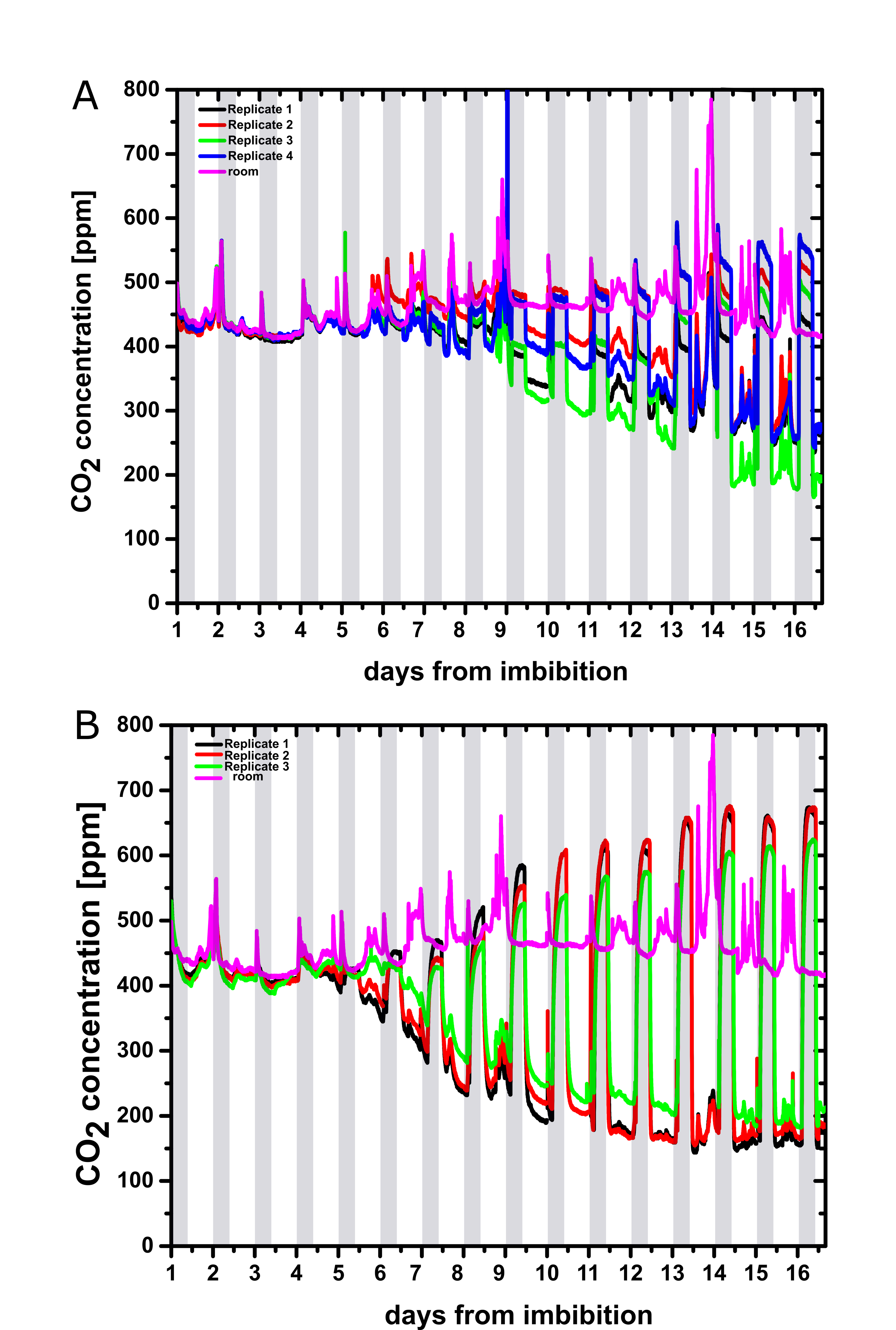

Supplement: S6 Fig — A. Micropore-sealed Petri dishes. B. Parafilm-sealed Petri dishes with 15 plants each (dark period shown in gray) with multiple replicates. (TIF) [file pone.0212462.s006.tif]

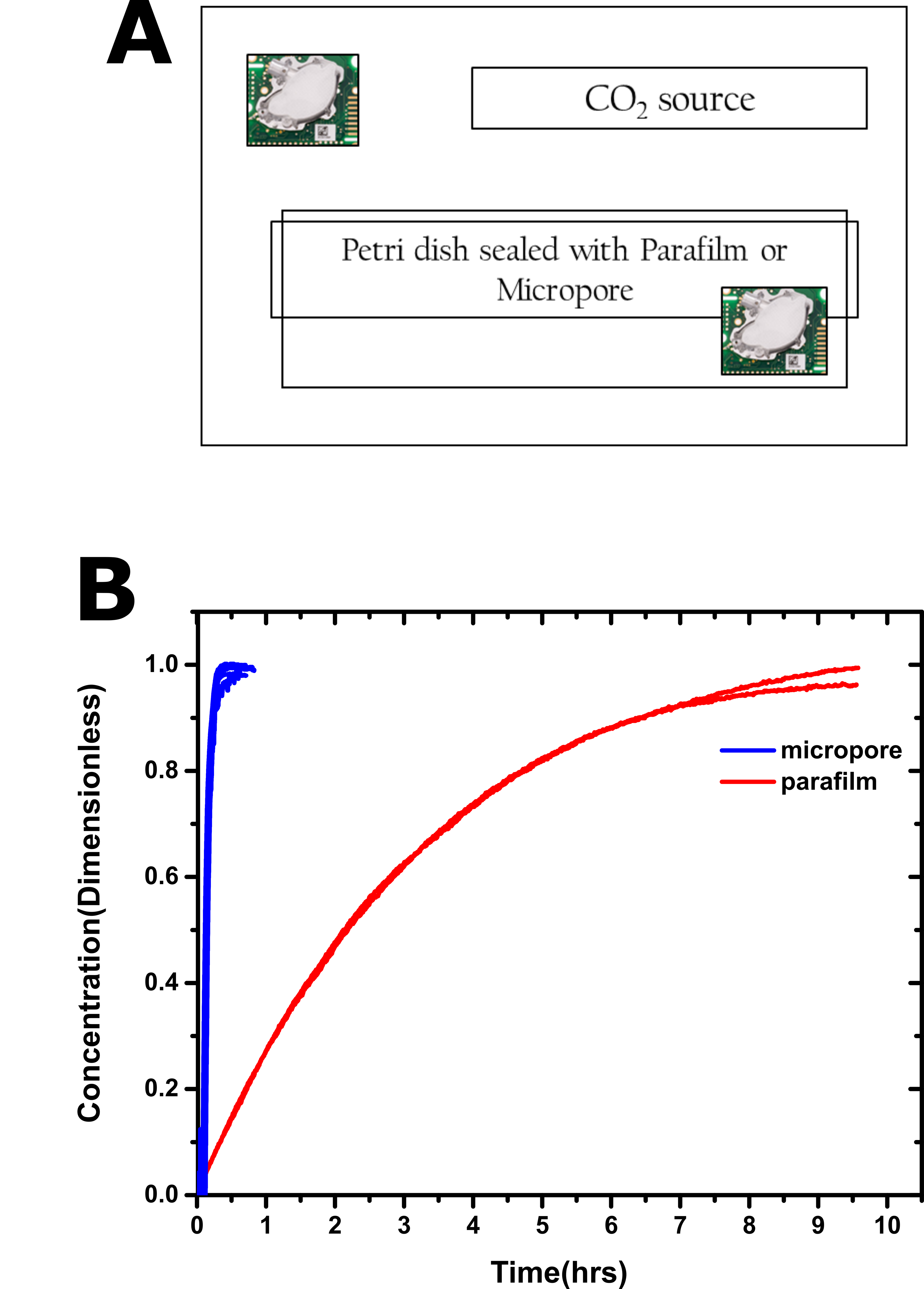

Supplement: S7 Fig — A. Experimental set up. B. Concentration–time plot for two different membranes. (TIF) [file pone.0212462.s007.tif]

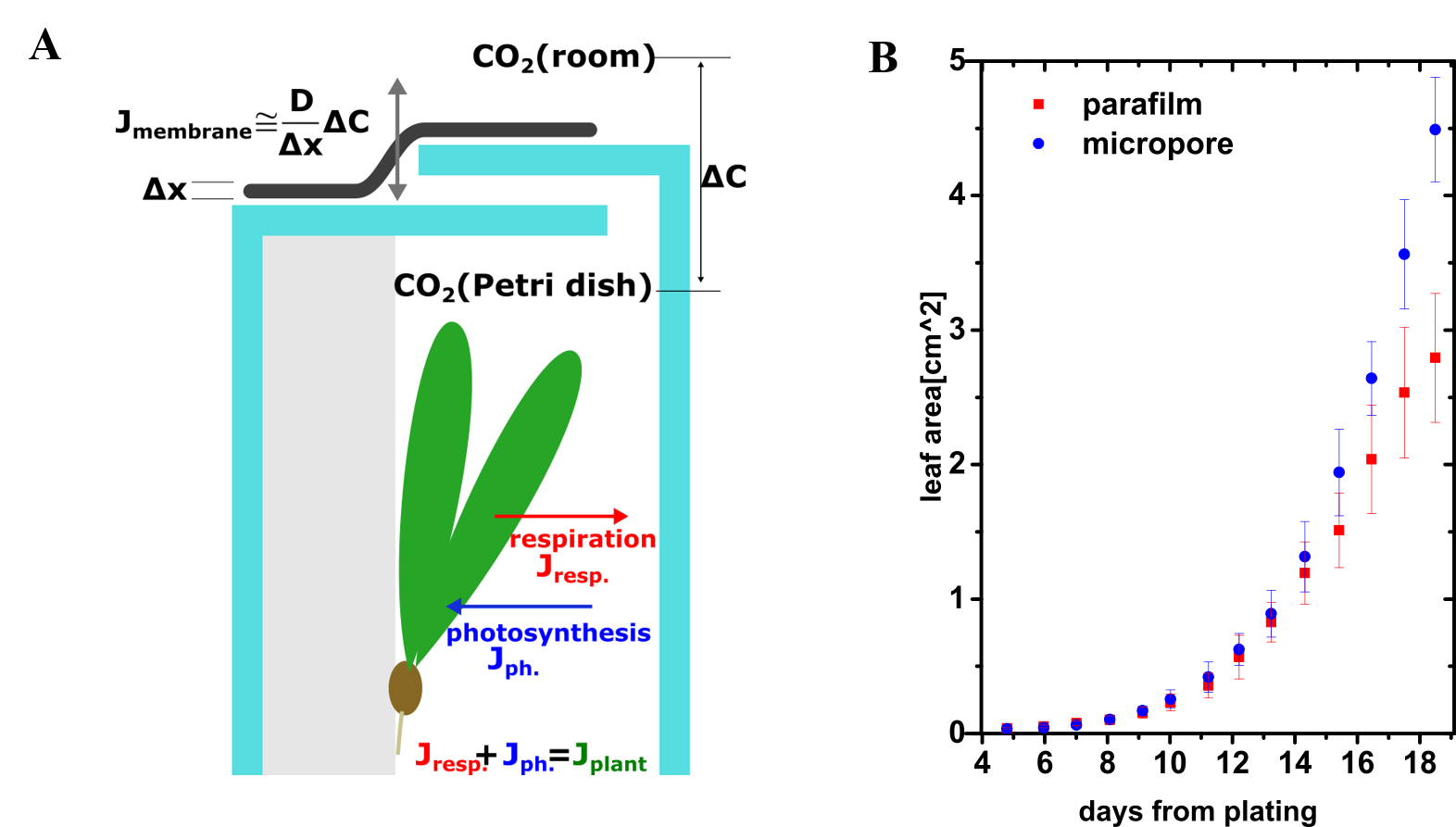

Supplement: S8 Fig — A Dynamics of CO2 exchange for plant cultures in Petri dishes. B Leaf growth dynamics over time for Parafilm and Micropore wrapped plant cultures with 15 plants. (TIF) [file pone.0212462.s008.tif]

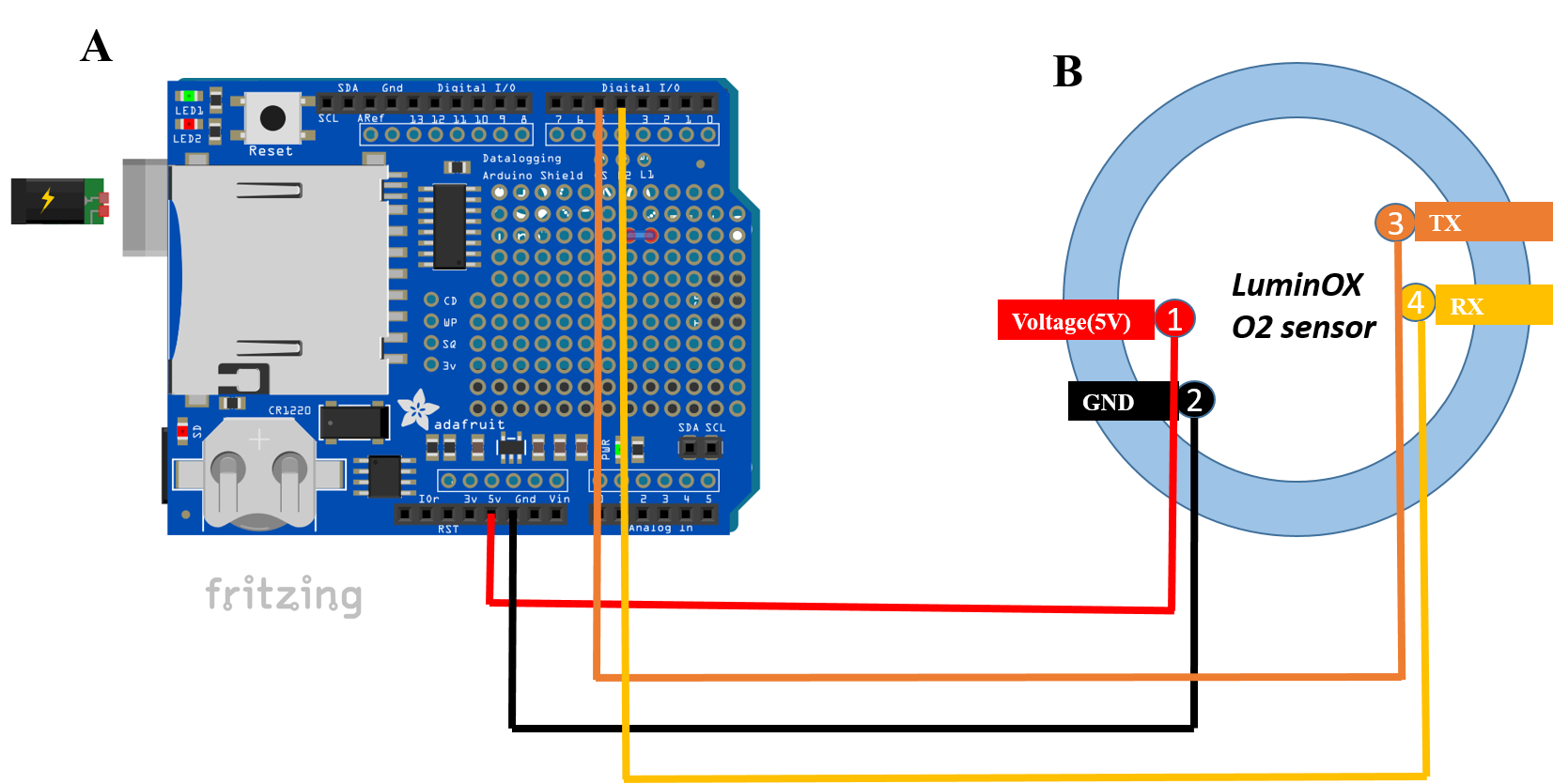

Supplement: S9 Fig — (TIF) [file pone.0212462.s009.tif]

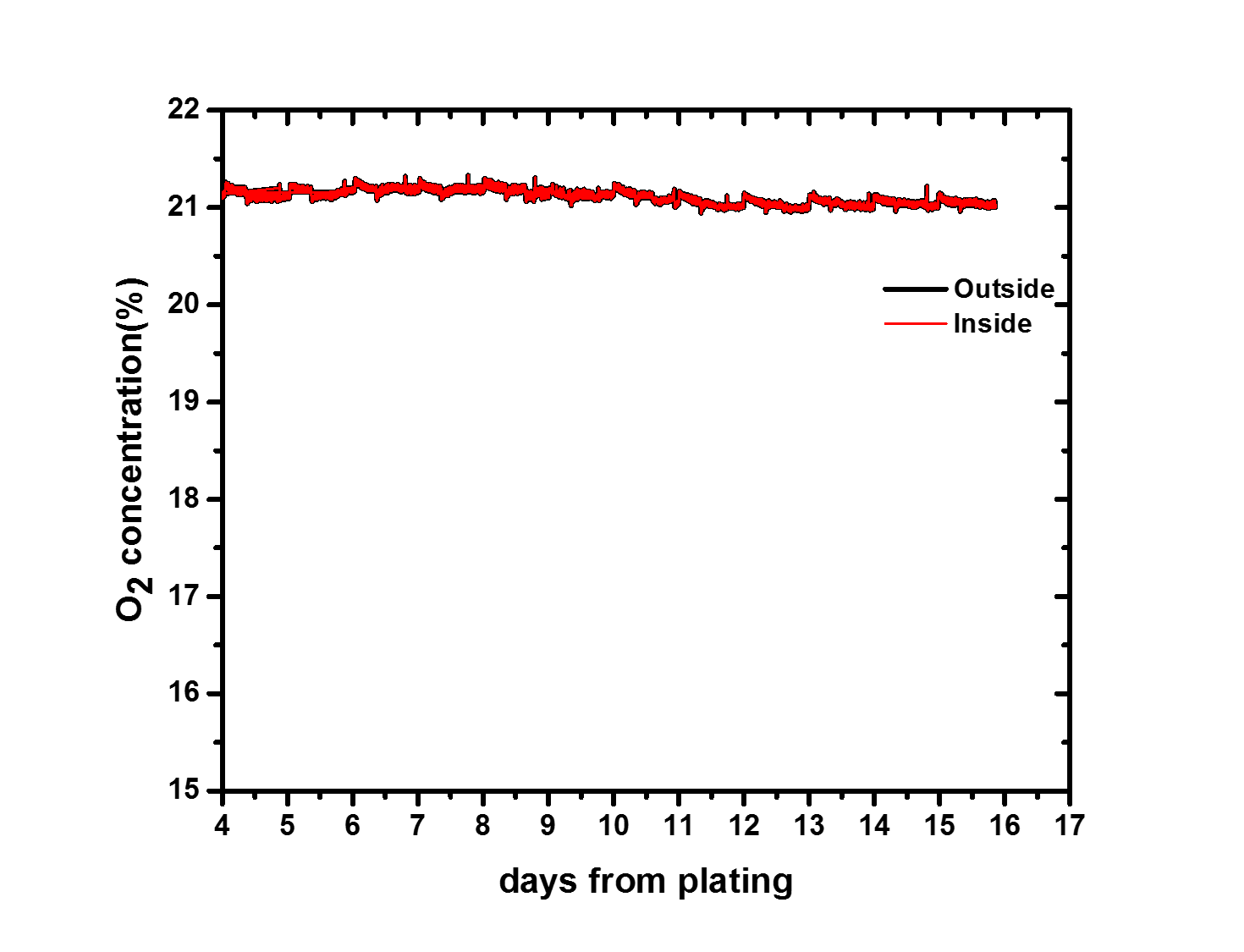

Supplement: S10 Fig — (TIF) [file pone.0212462.s010.tif]

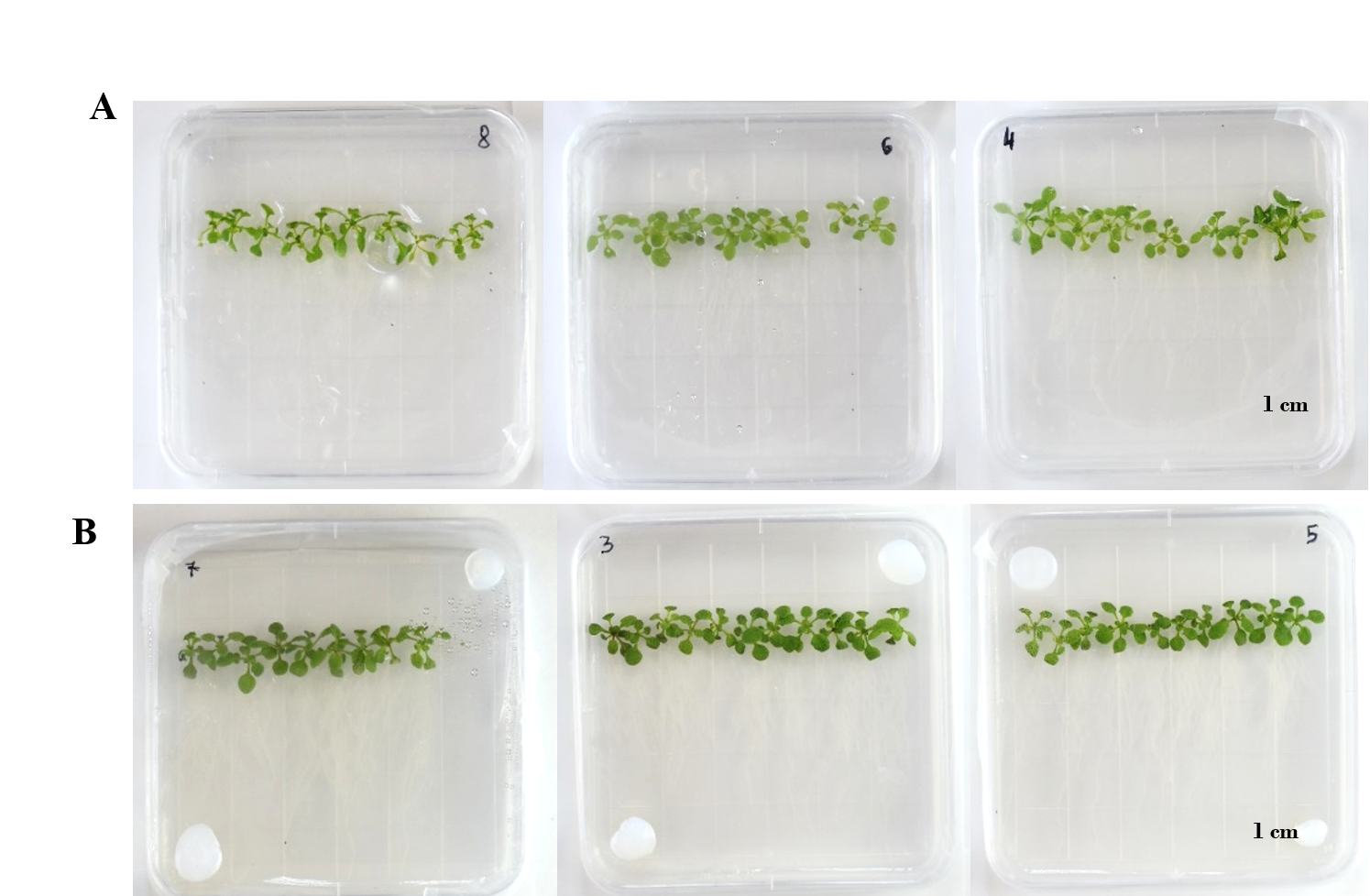

Supplement: S11 Fig — A and aerated B plant cultures after 2 weeks before extraction of RNA samples for gene expression. (TIF) [file pone.0212462.s011.tif]
