# Supplementary material for: Stress response to CO2 deprivation by Arabidopsis thaliana in plant cultures
Source: PLoS One. 2019 Mar 13;14(3):e0212462. doi: 10.1371/journal.pone.0212462 (PMC6415875; doi:10.1371/journal.pone.0212462)
Supplement: S1 Appendix — (DOCX) [file pone.0212462.s013.docx]

Stress response to CO_2_ deprivation by *Arabidopsis thaliana* in plant cultures

*Souvik Banerjee^1‡^, Oskar Siemianowski^1‡^, Meiling Liu^2,3^, Kara R. LIND^1^, Xinchun Tian^1^, Dan Nettleton^2^_,_ Ludovico Cademartiri^1,4,5*^*

*^1^ Department of Materials Science & Engineering, Iowa State University of Science and Technology, 2220 Hoover Hall, Ames, IA, 50011*

*^2^ Department of Statistics, Iowa State University of Science and Technology, 2438 Osborn Dr, Ames, IA, 50011-1090*

*^3^ Bioinformatics and Computational Biology Program, Iowa State University of Science and Technology, 2014 Molecular Biology Building, Ames IA 50011*

*^4^ Department of Chemical & Biological Engineering, Iowa State University of Science and Technology, Sweeney Hall, Ames, IA, 50011*

*^5^* *Ames Laboratory, U.S. Department of Energy, Ames, IA, 50011*

*^‡^ These authors contributed equally to the work*

*^*^ Author to whom correspondence should be addressed: lcademar@iastate.edu*

Appendix

**This PDF file includes:**

Arduino sketches for sensors (page 3 to 15).

R code for DESeq analysis (page 16).

Arduino sketches for sensors

**1: C++ code for CO_2_ measurement with K-30 sensor**

#include <SD.h>

#include <Wire.h>

#include <SPI.h>

#include "RTClib.h"

#include "kSeries.h"

// Create K30 instance on pin 4 & 5

kSeries K_30(4,5);

unsigned long time;

#if defined(ARDUINO_ARCH_SAMD)

// for Zero, output on USB Serial console, remove line below if using programming port to program the Zero!

#define Serial SerialUSB

#endif

// how many milliseconds between grabbing data and logging it. 1000 ms is a second

#define LOG_INTERVAL 1000 // mills between entries (reduce to take more/faster data)

// how many milliseconds before writing the logged data permanently to disk

// set it to the LOG_INTERVAL to write each time (safest)

// set it to 10*LOG_INTERVAL to write all data every 10 datareads, you could lose up to

// the last 10 reads if power is lost but it uses less power and is much faster!

#define SYNC_INTERVAL 1000 // mills between calls to flush() - to write data to the card

uint32_t syncTime = 0; // time of last sync()

#define ECHO_TO_SERIAL 1 // echo data to serial port

#define WAIT_TO_START 0 // Wait for serial input in setup()

// the digital pins that connect to the LEDs

#define redLEDpin 2

#define greenLEDpin 3

RTC_DS1307 RTC; // define the Real Time Clock object

// for the data logging shield, we use digital pin 10 for the SD cs line

const int chipSelect = 10;

// the logging file

File logfile;

void error(char *str)

{

Serial.print("error: ");

Serial.println(str);

// red LED indicates error

digitalWrite(redLEDpin, HIGH);

while(1);

}

void setup()

{

Serial.begin(9600); // the bigger number the better

Serial.println();

// use debugging LEDs

pinMode(redLEDpin, OUTPUT);

pinMode(greenLEDpin, OUTPUT);

#ifndef ESP8266

while (!Serial); // will pause Zero, Leonardo, etc until serial console opens

#endif

// wait for MAX chip to stabilize

delay(500);

#if WAIT_TO_START

Serial.println("Type any character to start");

while (!Serial.available());

#endif //WAIT_TO_START

// initialize the SD card

Serial.print("Initializing SD card...");

// make sure that the default chip select pin is set to

// output, even if you don't use it:

pinMode(10, OUTPUT);

// see if the card is present and can be initialized:

if (!SD.begin(chipSelect)) {

error("Card failed, or not present");

}

Serial.println("card initialized.");

// create a new file

char filename[] = "LOG0000.CSV";

for (uint16_t i = 0; i < 10000; i++) {

filename[3] = i/1000 + '0';

filename[4] = (i%1000)/100 + '0';

filename[5] = (i%100)/10 + '0';

filename[6] = i%10 + '0';

if (! SD.exists(filename)) {

// only open a new file if it doesn't exist

logfile = SD.open(filename, FILE_WRITE);

break; // leave the loop!

}

}

if (! logfile) {

error("couldnt create file");

}

Serial.print("Logging to: ");

Serial.println(filename);

// connect to RTC

Wire.begin();

if (!RTC.begin()) {

logfile.println("RTC failed");

#if ECHO_TO_SERIAL

Serial.println("RTC failed");

#endif //ECHO_TO_SERIAL

}

Serial.println("CLEARDATA"); //clears up any data left from previous projects

Serial.println("LABEL,time,timer, CO2(ppm)"); //always write LABEL, so excel knows the next things will be the names of the columns (instead of Acolumn you could write Time for instance)

Serial.println("RESETTIMER"); //resets timer to 0

logfile.println("millis,stamp,datetime,temp");

#if ECHO_TO_SERIAL

Serial.println("millis,stamp,datetime,temp");

#endif //ECHO_TO_SERIAL

}

void loop()

{

// Get CO2 value from sensor

double co2 = K_30.getCO2('p');

DateTime now;

// delay for the amount of time we want between readings

delay((LOG_INTERVAL -1) - (millis() % LOG_INTERVAL));

digitalWrite(greenLEDpin, HIGH);

// log milliseconds since starting

uint32_t m = millis();

logfile.print((m+2)/1000); // seconds since start

logfile.print("\t");

#if ECHO_TO_SERIAL

Serial.print((m+2)/1000); // seconds since start

Serial.print("\t");

#endif

// fetch the time

now = RTC.now();

// log time

logfile.print(now.year(), DEC);

logfile.print("/");

logfile.print(now.month(), DEC);

logfile.print("/");

logfile.print(now.day(), DEC);

logfile.print("\t");

logfile.print(now.hour(), DEC);

logfile.print(":");

logfile.print(now.minute(), DEC);

logfile.print(":");

logfile.print(now.second(), DEC);

logfile.print('\t');

#if ECHO_TO_SERIAL

Serial.print(now.year(), DEC);

Serial.print("/");

Serial.print(now.month(), DEC);

Serial.print("/");

Serial.print(now.day(), DEC);

Serial.print("\t");

Serial.print(now.hour(), DEC);

Serial.print(":");

Serial.print(now.minute(), DEC);

Serial.print(":");

Serial.print(now.second(), DEC);

Serial.print('\t');

#endif //ECHO_TO_SERIAL

// logfile.println("DATA,TIME,TIMER,");

logfile.print("CO2 ppm = ");

logfile.print(co2);

logfile.print("\t");

logfile.print("Time = ");

logfile.println(millis()/1000);

#if ECHO_TO_SERIAL

// Serial.println("DATA,TIME,TIMER,"); //writes the time in the first column A and the time since the measurements started in column B

Serial.print("CO2 ppm = ");

Serial.print(co2); // Print the sensor value on Serial

Serial.print("\t");

Serial.print("Time = ");

Serial.println(millis()/1000);//prints time since program started

#endif //ECHO_TO_SERIAL

logfile.println();

#if ECHO_TO_SERIAL

Serial.println();

#endif // ECHO_TO_SERIAL

// Wait 10 seconds

delay(500);

digitalWrite(greenLEDpin, LOW);

// Now we write data to disk! Don't sync too often - requires 2048 bytes of I/O to SD card

// which uses a bunch of power and takes time

if ((millis() - syncTime) < SYNC_INTERVAL) return;

syncTime = millis();

// blink LED to show we are syncing data to the card & updating FAT!

digitalWrite(redLEDpin, HIGH);

logfile.flush();

digitalWrite(redLEDpin, LOW);

}

**2: C++ code for O_2_ measurement with Luminox oxygen sensor**

#include <SD.h>

#include <Wire.h>

#include <SPI.h>

#include "RTClib.h"

#include <SoftwareSerial.h> //incorporate software-serial libray

SoftwareSerial myserial(4, 5); //enable software serial port 10 to RX, and 11 to TX

String Luminoxstring = ""; //string to hold incoming data from Luminox-O2 sensor

boolean Luminox_stringcomplete = false;

long previousMillis = 0;

unsigned long time;

#if defined(ARDUINO_ARCH_SAMD)

// for Zero, output on USB Serial console, remove line below if using programming port to program the Zero!

#define Serial SerialUSB

#endif

#define LOG_INTERVAL 1000 // mills between entries (reduce to take more/faster data)

#define SYNC_INTERVAL 1000 // mills between calls to flush() - to write data to the card

uint32_t syncTime = 0; // time of last sync()

#define ECHO_TO_SERIAL 1 // echo data to serial port

#define WAIT_TO_START 0 // Wait for serial input in setup()

// the digital pins that connect to the LEDs

#define redLEDpin 2

#define greenLEDpin 3

RTC_DS1307 RTC; // define the Real Time Clock object

// for the data logging shield, we use digital pin 10 for the SD cs line

const int chipSelect = 10;

// the logging file

File logfile;

//were all data from Luminox-O2 sensor received? check

void error(char *str)

{

Serial.print("error: ");

Serial.println(str);

// red LED indicates error

digitalWrite(redLEDpin, HIGH);

while(1);

}

void setup()

{

Serial.begin(9600); //set baud rate for Arduino serial port to 9600

myserial.begin(9600); //set baud rate for software serial port to 9600

Luminoxstring.reserve(41);

Serial.print("Initializing SD card...");

// make sure that the default chip select pin is set to

// output, even if you don't use it:

pinMode(10, OUTPUT);

// see if the card is present and can be initialized:

if (!SD.begin(chipSelect)) {

Serial.println("Card failed, or not present");

// don't do anything more:

return;

}

Serial.println("card initialized.");

// create a new file

char filename[] = "LOG0000.CSV";

for (uint16_t k = 0; k < 10000; k++) {

filename[3] = k/1000 + '0';

filename[4] = (k%1000)/100 + '0';

filename[5] = (k%100)/10 + '0';

filename[6] = k%10 + '0';

if (! SD.exists(filename)) {

// only open a new file if it doesn't exist

logfile = SD.open(filename, FILE_WRITE);

break; // leave the loop!

}

}

if (! logfile) {

error("couldnt create file");

}

Serial.print("Logging to: ");

Serial.println(filename);

// connect to RTC

Wire.begin();

if (!RTC.begin()) {

logfile.println("RTC failed");

#if ECHO_TO_SERIAL

Serial.println("RTC failed");

#endif //ECHO_TO_SERIAL//set aside 41 bytes for receiving data from Luminox-O2 sensor

}

Serial.println("CLEARDATA"); //clears up any data left from previous projects

Serial.println("LABEL,time,timer, CO2(ppm)"); //always write LABEL, so excel knows the next things will be the names of the columns (instead of Acolumn you could write Time for instance)

Serial.println("RESETTIMER"); //resets timer to 0

logfile.println("millis,stamp,datetime,temp");

#if ECHO_TO_SERIAL

Serial.println("millis,stamp,datetime,temp");

#endif //ECHO_TO_SERIAL

}

void loop()

{

{ //start the loop sequence

while (myserial.available()) { //when a char is avaialable in software serial buffer

char inchar = (char)myserial.read();

// Serial.print(inchar); //grab that char

Luminoxstring += inchar; //add the received char to LuminoxString

if (inchar == '\r') { //if the incoming character is a <term>, reset

Luminox_stringcomplete = true; //then a complete string of data has been recieved from Luminox-O2 sensor

}

}

}if (Luminox_stringcomplete){ //has a complete string from the Luminox sensor has been received?

Luminoxstring.remove(41); //remove any serial string overruns between reads

Serial.print(Luminoxstring);

logfile.print(Luminoxstring);

//use the Arduino serial port to send that data to CoolTerm

Luminoxstring = ""; //then clear the Luminoxstring:

Luminox_stringcomplete = false; //await the next data string from Luminox-O2 sensor

}

DateTime now;

// delay for the amount of time we want between readings

delay((LOG_INTERVAL -1) - (millis() % LOG_INTERVAL));

digitalWrite(greenLEDpin, HIGH);

//digitalWrite(greenLEDpin, HIGH);

// log milliseconds since starting

uint32_t m = millis();

logfile.print((m+2)/1000); // seconds since start

logfile.print("\t");

#if ECHO_TO_SERIAL

Serial.print((m+2)/1000); // seconds since start

Serial.print("\t");

#endif

// fetch the time

now = RTC.now();

// log time

logfile.print(now.second(), DEC);

logfile.print('\t');

#if ECHO_TO_SERIAL

Serial.print(now.second(), DEC);

Serial.print('\t');

#endif //ECHO_TO_SERIAL

// logfile.println("DATA,TIME,TIMER,");

logfile.print("\t");

logfile.print("Time = ");

logfile.println(millis()/1000);

#if ECHO_TO_SERIAL

// Serial.println("DATA,TIME,TIMER,"); //writes the time in the first column A and the time since the measurements started in column B

Serial.print("\t");

Serial.print("Time = ");

Serial.print(Luminoxstring);

Serial.println(millis()/1000);//prints time since program started

#endif //ECHO_TO_SERIAL

logfile.println();

#if ECHO_TO_SERIAL

Serial.println();

#endif // ECHO_TO_SERIAL

// Wait 10 seconds

// delay(500);

//digitalWrite(greenLEDpin, LOW);

// Now we write data to disk! Don't sync too often - requires 2048 bytes of I/O to SD card

// which uses a bunch of power and takes time

if ((millis() - syncTime) < SYNC_INTERVAL) return;

syncTime = millis();

//blink LED to show we are syncing data to the card & updating FAT!

digitalWrite(redLEDpin, HIGH);

logfile.flush();

digitalWrite(redLEDpin, LOW);

}

**R code for DESeq analysis**

*# Get biocunductor and call the DEseq2 library*

source("http://bioconductor.org/biocLite.R")

biocLite("DESeq2")

library("DESeq2")

*# set the working directory to the relevant folder*

setwd("~/LC_gene_counts/")

dat<-read.table("At.combined.gene.txt",header = T,quote = "",row.names = 1, col.names=c(

*# Convert to matrix*

dat <- as.matrix(dat)

head(dat)

*# Assign conditions (Aerated: AER, Non-Aerated: NON)*

condition <- factor(rep(c("AER","NON"),4))

condition=relevel(condition,**ref** = "NON")

*# Create a coldata frame: its rows correspond to columns of dat (i.e., matrix representing the countData)*

coldata <- data.frame(row.names=colnames(dat), condition)

coldata

*#DESEq pipeline, first make the design and then normalizing and model fitting*

dds <- DESeqDataSetFromMatrix(countData = dat, colData = coldata,design=~ condition)

dds <- DESeq(dds)

*# Plot Dispersions:*

png("qc-dispersions.png", 1000, 1000, pointsize=20)

plotDispEsts(dds, main="Dispersion plot")

dev.off()
